# Genome-wide characterization and metabolite profiling of *Cyathus olla*: insights into the biosynthesis of medicinal compounds

**DOI:** 10.1186/s12864-024-10528-3

**Published:** 2024-06-19

**Authors:** Xiuchao Xie, Ling Zhao, Yu Song, Yanming Qiao, Zhen-Xin Wang, Jianzhao Qi

**Affiliations:** 1 Shaanxi Province Key Laboratory of Bio-resources, Qinba State Key Laboratory of Biological Resources and Ecological Environment (Incubation), School of Biological Science and Engineering, Shaanxi University of Technology, Hanzhong, 723000 China; 2 Department of Pharmacy, School of Medicine, Xi’an International University, Xi’an 710077, China; 3 Shaanxi Key Laboratory of Natural Products & Chemical Biology, College of Chemistry & Pharmacy, Northwest A&F University, Yangling, 712100 China

**Keywords:** Chromosome-level assembly, Comparative genomic, *Cyathus olla*, Medicinal fungi

## Abstract

*Cyathus olla*, belonging to the genus *Cyathus* within the order Agaricales, is renowned for its bird's nest-like fruiting bodies and has been utilized in folk medicine. However, its genome remains poorly understood. To investigate genomic diversity within the genus *Cyathus* and elucidate biosynthetic pathways for medicinal compounds, we generated a high-quality genome assembly of *C. olla* with fourteen chromosomes. The comparative genome analysis revealed variations in both genomes and specific functional genes within the genus *Cyathus*. Phylogenomic and gene family variation analyses provided insights into evolutionary divergence, as well as genome expansion and contraction in individual *Cyathus* species and 36 typical Basidiomycota. Furthermore, analysis of LTR-RT and Ka/Ks revealed apparent whole-genome duplication (WGD) events its genome. Through genome mining and metabolite profiling, we identified the biosynthetic gene cluster (BGC) for cyathane diterpenes from *C. olla*. Furthermore, we predicted 32 BGCs, containing 41 core genes, involved in other bioactive metabolites. These findings represent a valuable genomic resource that will enhance our understanding of *Cyathus* species genetic diversity. The genome analysis of *C. olla* provides insights into the biosynthesis of medicinal compounds and establishes a fundamental basis for future investigations into the genetic basis of chemodiversity in this significant medicinal fungus.

**Supplementary Information:**

The online version contains supplementary material available at 10.1186/s12864-024-10528-3.

## Introduction

Mushrooms, colloquially referred to as large fungi capable of forming specific fruiting bodies, have been a significant source of food and medicine for thousands of years due to their unique nutritional value and medicinal properties [1]. As the significance of medicinal mushrooms for human health increases and the popularity of sequencing technology grows, researchers are sequencing and decoding the genomes of an ever-increasing number of valuable medicinal mushrooms. The deciphering of these genomes enables a more comprehensive understanding of their mating types, nutritional models, medicinal ingredient biosynthesis, high-yield cultivation and population genetics research, thereby further promoting the research of their medicinal value and the development of the health industry. *Ganoderma lucidum* is one of the first reported medicinal mushrooms to have its genome sequenced [2]. This achievement has facilitated promoting the elucidation of the biosynthesis of medicinal ingredients in *Ganoderma* mushrooms and their cultivation. This work initiated a research frenzy in the genome sequencing of medicinal mushrooms. Subsequently, the genomes of several rare medicinal mushrooms including *Laetiporus sulphureus* [3], *Antrodia cinnamomea* [4], *Inonotus hispidus* [5], and *Inonotus obliquus* [6], have been sequenced and reported.

The term “bird's nest fungi” is used to collectively reference to a group of fungi that exhibit a distinctive shape and are classified within the family Nidulariaceae [7, 8]. Despite belonging to the order Agaricales, the bird's nest appearance of these fungi distinguishes them significantly from other members of the umbrella-shaped Agaricales. The mechanisms and evolutionary divergence that give rise to their distinctive appearance have been the subject of considerable attention [8, 9]. The genus *Cyathus* is the most extensively studied medicinal mushroom within the family Nidulariaceae. *Cyathus helenae* H.J. Brodie, *Cyathus stercoreus* (Schwein.) De Toni, and *Cyathus striatus* (Huds.) Willd. are three documented medicinal fungi found in classic traditional Chinese medicine literatures [10]. They have been documented to possess antibacterial and antifungal properties, and can be employed in the treatment of gastropathy [10]. Cyathane diterpenoids are structurally distinct secondary metabolites discovered in the genus *Cyathus* [11–13], and their most notable activity is for their anti-neurodegenerative properties. In addition, these compounds have been investigated for their potential anti-cancer effects [14, 15].

**Fig. 1:**
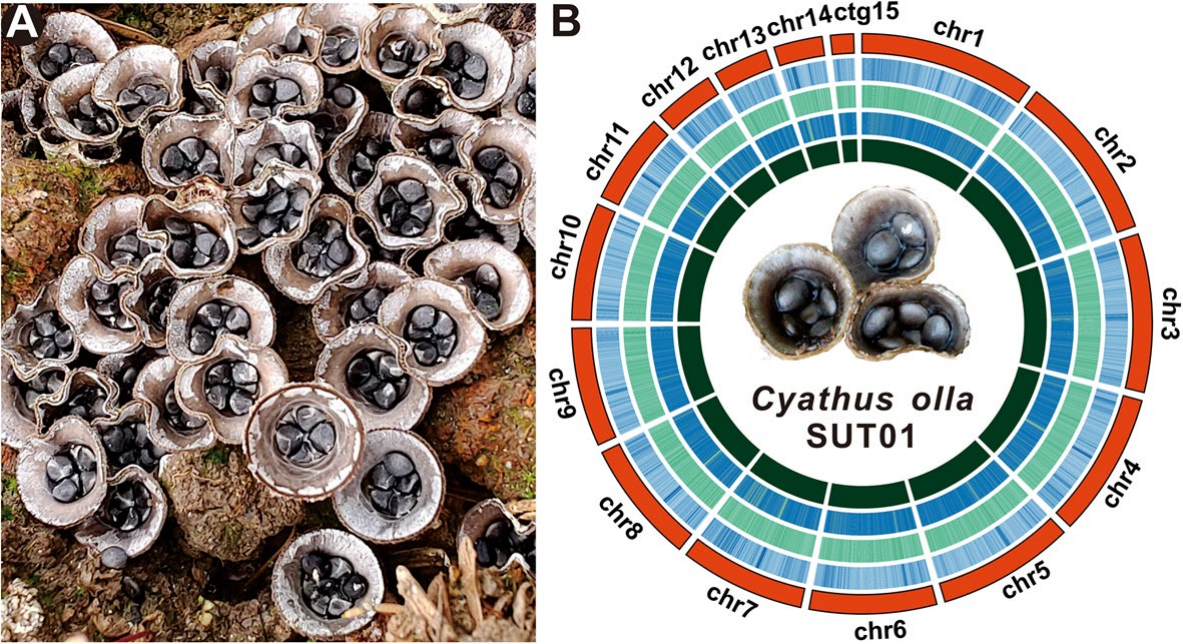
Morphological and genomic features of *Cyathus olla*. **A** The fruiting body of *Cyathus olla* growing in patches, **B** Genomic characterization of *Cyathus olla*. From the outside to the inside are I. Chromosome and Contigs; II–IV. GC-density, GC-skew, AT-skew (window size 1 kb), V. Gene-density (window size 1kb)

*Cyathus olla* is an unusual bird's nest fungus. Few studies have found its potential as an antimicrobial agent to reduce the incidence of stubble diseases in oilseed rape [16, 17]. *Cyathus olla* inhabited the Qinba Mountains has been used as a classic folklore medicine in local for thousands of years. A patch of *C. olla* (Fig. 1A), located in Zhenba County, was collected in 2019 under the guidance of an old herbalist. Morphological character and ITS alignment (Supplementary Fig. S1) confirmed the species, which was named *Cyathus olla* SUT1. Its crude extract exhibited significant NGF-dependent promotional activity on rat phaeochromocytoma PC12 cells (Supplementary Fig. S2). In light of the considerable medicinal potential attributed to *C. olla*, coupled with the dearth of genomic information pertaining to this species, the present study marks a pioneering effort in achieving a chromosome-level genome assembly of *C. olla*. This was accomplished through the synergistic application of Nanopore PromethION and Illumina NovaSeq sequencing technologies, which are at the forefront of genomic research. The resultant high-fidelity genomic sequence has been instrumental in our comprehensive examination of *C. olla*'s phylogenetic positioning. Notably, this investigation has led to the discovery of a BGC responsible for the production of cyathane diterpenes, a class of compounds with significant medicinal implications. Furthermore, the identification of several candidate genes implicated in the biosynthesis of secondary metabolites underscores the study's contribution to the field. Our comparative genomic analysis has unveiled intriguing variations within the genome of *C. olla* and the broader genus *Cyathus*. These findings, coupled with gene family variation analysis, have shed light on the genomic expansion and reduction phenomena observed in both *C. olla* and representative species of Basidiomycota. Such insights are pivotal for understanding the evolutionary dynamics at play within these fungal lineages.

## Materials and methods

### Fungal material, extraction of genomic DNA and RNA

The strain of *C. olla* UST01 was initially isolated from Zhenba County, Shaanxi Province. Tissue isolation was carried out from sterile fresh fruiting bodies to obtain strain, and the confirmed strain were deposited in the form of slants in Shaanxi Key Laboratory of Natural Products and Chemical Biology, Northwest A&F University. The mycelial culture of *C. olla* UST01 was carried out in Potato Dextrose Broth (PDB) medium at 25 ℃ with an agitation rate of 250 rpm for ten days. Genomic DNA was extracted from the mycelium using the sodium dodecyl sulphate method, and the specific isolation and purification method were described in a previous literature [18, 19]. RNAs were extracted from mycelium using a RNeasy Plant Mini Kit (Qiagen, Netherlands) following the manufacturer’s protocol.

### Genome sequencing, *de novo* assembly, annotation, and visualization

#### Library construction and sequencing

After assessing the quality and integrity of the DNA, it was randomly fragmented using a Covaris ultrasonic disruptor (Covaris, Woburn, MA, USA). Pair-end Illumina sequencing libraries were then constructed using the Nextera DNA Flex Library Prep Kit (Illumina, San Diego, CA, USA) with an insert size of 300 bp. Sequencing was performed on the Illumina NovaSeq6000 platform (Illumina, San Diego, CA, USA). To ensure high quality data, the raw reads underwent a cleaning process to remove low quality reads. This was done using SOAPnuke v2.1.8 (https://github.com/BGI-flexlab/SOAPnuke). The clean data was then used for subsequent analyses. For Oxford Nanopore sequencing, libraries were prepared using the SQK-LSK109 ligation kit (Oxford Nanopore Technologies, Oxford, UK) according to the standard protocol. The purified library was then loaded onto primed R9.4 Spot-On Flow Cells and sequenced using a PromethION sequencer (Oxford Nanopore Technologies, Oxford, UK). Base calling analysis was performed using Oxford Nanopore GUPPY v6.4.6 (https://community.nanoporetech.com/downloads) to obtain sequence data from the raw reads. All of the above softwares run according to the default parameters.

#### *De Novo* Assembly

The genome size of *C. olla* SUT01 was estimated with Genomescope 2.0 (https://github.com/tbenavi1/genomescope2.0) using the Illumina reads. The NECAT software (https://github.com/xiaochuanle/NECAT) was used for genome correction and assembly, resulting in an initial assembly. This initial assembly underwent two rounds of error correction using Racon V1.5 (https://github.com/isovic/racon) with default parameters, using nanopore reads. Two further rounds of error correction were then performed using Pilon 1.2.4 (https://github.com/broadinstitute/pilon) with default parameters, this time using second-generation reads. Finally, the corrected genome was further processed using purge_haplotigs to eliminate heterozygous sequences, resulting in the final assembled genome.

#### Gene prediction and annotation

BRAKER v2.1.4 (https://github.com/Gaius-Augustus/BRAKER) was used primarily for gene prediction. The model was trained using GeneMark-EX and open reading frames (ORFs) were predicted using AUGUSTUS V3.3.3 (https://github.com/Gaius-Augustus/Augustus). INFERNAL v1.1.2 (https://github.com/EddyRivasLab/infernal) was used to predict and categorize non-coding RNAs with the Rfam 14.6 database. Finally, BLAST searches of non-redundant protein sequences from databases such as NCBI, Swiss-Prot, COG and KEGG were performed for gene product annotation.

#### Genomic circle map

The genomic circle map was analyzed and visualized using McscanX (https://github.com/wyp1125/MCScanX), running with default parameters.

### Comparative genomics analysis

The completeness of the gene set was assessed with Compleasm v0.25 (https://github.com/huangnengCSU/compleasm), using the database fungi_odb10. Clustering analysis of the comparative genomes of the genus *Cyathus* species was performed using Orthofinder v2.5.5 (https://github.com/davidemms/OrthoFinder), which run using the following settings: -S diamond -M msa -T fasttree -t 128. The comparative genome results were visualized using jVenn (http://jvenn.toulouse.inra.fr/app/index.html). The genes encoding carbohydrate-active enzymes (CAZymes) of four *Cyathus* species were annotated and classified using the CAZy database (http://bcb.unl.edu/dbCAN2) via HMMER package (V 3.2.1, filter parameter E-value < 1e^−18^; coverage > 0.35). Prediction of biosynthetic gene clusters (BGCs) of four *Cyathus* species was accomplished with the fungal version of antiSMASH 7.0 (https://fungismash.secondarymetabolites.org), configuring the detection strictness as relaxed and extra features parameter as all on

### Phylogenomic analysis and gene family variation analysis

A phylogenetic analysis was performed to investigate the evolutionary relationships between *Cyathus* species and 36 other representative species of Basidiomycetes. Single-copy homologous genes were identified using OrthoFinder v2.5.5 with the parameters "-S diamond -M msa -T raxml-ng". Divergence time prediction of 236 single-copy orthologous sequences from 40 strains was performed using MCMCTree within PAML 4.9e (http://abacus.gene.ucl.ac.uk/software/paml.html). The divergence times of several groups of recent ancestors were analyzed using TIMETREE 5 (http://www.timetree.org). These groups included *Ganoderma sinense* vs *Grifola frondosa* (84.2–135.2 million years ago (MYA)), and *Laetiporus sulphureus* vs. *Gelatoporia subvermispora* (137.2–164.3 MYA), *Lyophyllum decastes* vs *Tricholoma matsutake* (90.6–118.1 MYA) and *Paxillus involutus* vs *Suillus brevipes* (90.2–130.0 MYA). To determine gene family expansion and contraction, CAFÉ 4.2.1 (https://github.com/hahnlab/CAFE) was utilized with identified orthologous gene families. The analysis was conducted with the following parameters: --cores 30 --fixed_lambda 0.0001.

### Transposon element, LTR-RT analysis, and WGD analysis

RepeatModeler v1.0.4 (https://github.com/Dfam-consortium/RepeatModeler) was used to generate a repeat library incorporating the Rebase library, and RepeatMasker v4.0.5 (https://github.com/rmhubley/RepeatMasker) was used to annotate repetitive genomic sequences. Long terminal repeat insertion times (LTR-RT) were identified and analyzed with the help of LTR_retriever v2.9.0 (https://github.com/oushujun/LTR_retriever).

To calculate the ratio of synonymous substitution rates (Ks) to nonsynonymous substitution rates (Ka) for each species of the genus *Cyathus*, genome-wide replication analyses were performed using wgd v1.1.2 (https://github.com/arzwa/wgd) and Para AT v2.0 (https://ngdc.cncb.ac.cn/tools/paraat).

### Metabolites profiling of *C. olla* SUT01

One gram of substrate extract and one gram of mycelial fermentation product extract were subjected to high resolution liquid chromatography mass spectrometry (HR-LCMS) followed by molecular network analysis, respectively. HR-LCMS detection was performed using an AB Sciex TripleTOF 6600 mass spectrometer (AB Sciex, MA, USA) in positive ion modes. Molecular network analysis of HPLC-HRMS data of crude extracts was performed using GNPS (https://gnps.ucsd.edu) with default parameters. The molecular networks were visualized by Cytoscape 3.9.1. Five kg of mycelial cultures of rice were extracted for metabolite isolation and identification. The structural characterization of the monomeric compounds was accomplished by HRMS, MS/MS, and/or NMR spectroscopy, and the data were acquired via a Bruker Avance III 500 MHz NMR spectrometer, using TMS as an internal standard, with chemical shifts recorded in parts per million *δ*(ppm).

### BGC analysis and visualization

The assessment of homology and similarity between two BGCs for cyathane diterpene was performed using Clinker [20], and visualization of the comparison results was achieved using clustermap.js [20], a tool embedded in Clinker to generate BGC comparison plots. Evolutionary tree-based cluster analysis is implemented via IQtree 2.2.2.6 (https://github.com/iqtree/iqtree2) with the options “-m MFP -bb 1000 -alrt 1000 -abayes -nt AUTO”. For a detailed analysis of multi-domain synthases such as NRPS and PKS, the package Synthaser [21] was used to analyze their domain characteristics. These domains include adenylation (A), acyl carrier protein (ACP), acyltransferase (AT), thiolation (T), thioesterase (TE), condensation (C), *β*-ketoacyl synthetase (KS), product template (PT), acyl carrier protein transacylase (SAT), and thioester reductase (TR).

## Results and discussion

### High-quality genome assembly and annotation of *Cyathus olla*

A total of 6.00 Gb Illumina NovaSeq clean base (Supplementary Table S1), and 6.41 Gb Nanopore PromethION pass data (Supplementary Tables S2), were used for assembly, resulting in a 47.83 Mb genome (Fig. 1B and Table 1), The genome comprised of fourteen chromosomal pseudomolecules and a contig, with an N50 of 3,637,865 bp and a 43.73% GC content (Supplementary Table S3). The K-mer curve showed two peaks with a more than three-fold difference in peak height, suggesting a heterozygosity of 5.97% for *C. olla* SUT01 (Supplementary Fig. S3), which indicated that it is a dikaryon. The high quality and completeness of the *C. olla* SUT1 genome assembly were supported by a 90.03% map rate, an average depth of 106.55x, and 99.91% coverage (Supplementary Table S4). To the best of our knowledge, this represents the first chromosome-level assembly of the *C. olla* genome.

**Table 1:** Comparison of sequencing metrics, and genome quality of four *Cyathus* Species

**entry**	***Cyathus olla*** **SUT01**	***Cyathus pallidus*** **NK-01**	***Cyathus stercoreus*** **NK-82**	***Cyathus striatus*** **AH 40144**
Sequencing technology	Illumina, Nanopore	PacBio	PacBio	Illumina, PacBio
Sequencing depth	106.55x	30.89x	53.76x	52.7x
No. of contig	1	235	421	1562
No. of chromosome	14	NA^a^	NA	NA
Total length (bp)	47,825,376	158,824,083	202,911,832	91,180,015
Largest length (bp)	5,034,100	7,510,000	6,000,000	2,130,000
Contig N50 (bp)	3,637,865	2,579,317	1,895,423	2,018,000
BUSCO completeness (%)	97.89	98.55	98.95	97.23
GC content (%)	43.73	44.88	45.83	45.50
No. of protein-coding genes	14,248	32,880	33,641	23,513
GenBank accession No.	SAMN40136088	SAMN30170265	SAMN30170247	GCA_015501535.1
References	This study	Kraisitudomsook *et. al* [9]	Kraisitudomsook *et. al* [9]	Kraisitudomsook *et. al* [9]

^a^*NA* Not available

The genome of *C. olla* SUT01 was predicted to have 14,248 protein-coding genes and 243 non-coding genes. The protein-coding genes had an average length of 1,272.37 base pairs (bp) and contained a total of 111,297 exons (with an average length of 174.94 bp) and 95,995 introns (with an average length of 74.68 bp) (Supplementary Table S5). The non-coding RNAs (ncRNAs) consisted of 205 tRNAs, 27 snRNAs, 10 rRNAs, and one sRNA (Supplementary Table S6). To achieve comprehensive functional annotation, transcriptome-based analysis (utilizing 1.50 Gb RNAseq data) and homology-based analysis using nine public databases (Nr, Pfam, eggNOG, UniProt, KEGG, GO, Pathway, RefSeq, and Interproscan, Supplementary Table S7) were combined. This resulted in the annotation of 14,248 protein-coding genes. Among these, the Nr database annotated 11,721 genes, accounting for 94.14% of the total. Interestingly, 53.44% of these genes showed similarities to the genome of Crucibulum laeve, a member of another genus in the family Nidulariaceae (Supplementary Fig. S4), indicating affinity within members of the family Nidulariaceae. Within the functional classification provided by the GO database, the "Biological_process" category constituted the largest group among the 6,216 annotated genes (Supplementary Fig. S5). A total of 1,261 genes were identified using the COG database, with 148 of them belonging to the I group, which is related to translation, ribosomal structure, and biogenesis (Supplementary Fig. S6). According to the KEGG database, 4,551 genes were associated with five types of pathways, with metabolic pathways having the largest number of genes (Supplementary Fig. S7). Analysis based on the Pfam database identified 8,867 genes containing various domains, with the top 20 domains shown in Supplementary Fig. S8. In conclusion, these findings show the functional diversity and characteristics of the protein-coding genes in the *C. olla* SUT1 genome. This study presents the first report on the genomic characterization of *C. olla*.

### Comparative genome analysis within *Cyathus* species

**Fig. 2:**
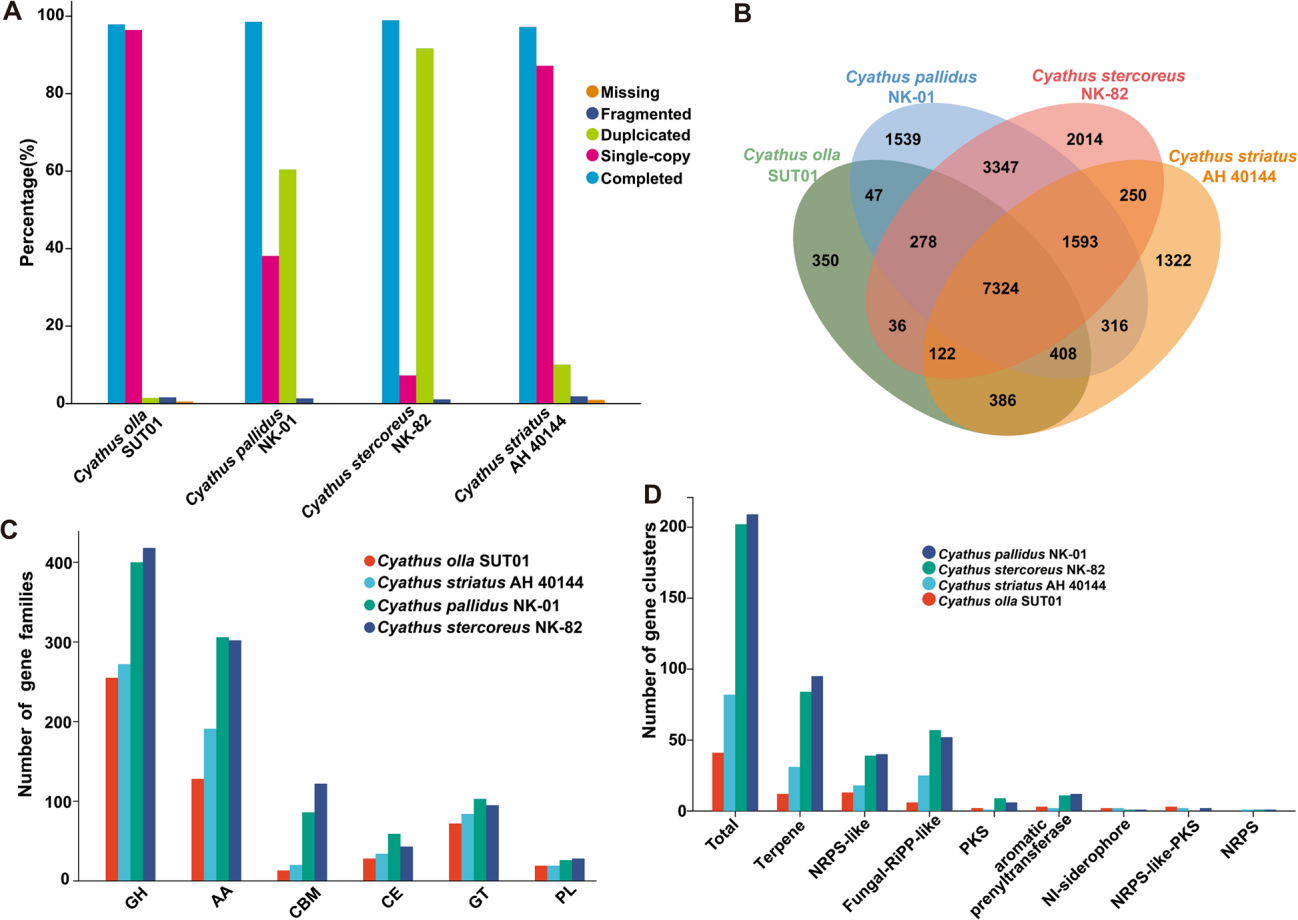
Comparative genomic analysis within the genus *Cyathus*. **A** Comparison of genome assembly quality evaluation based on the proportion of single-copy genes. **B** Venn diagram of unique and core gene families among the four *Cyathus* species. The number represents the number of gene families. **C** CAZyme-related gene families among the four Cyathus species. GH, glycoside hydrolase; AA, auxiliary activity; CBM, carbohydrate-binding module; CE, carbohydrate esterase; GT, glycosyl transferase; PL, polysaccharide lyase. **D** Secondary metabolite-related gene clusters in four *Cyathus* species. NRPS, non-ribosomal peptide synthetase; RIPP, Ribosomally synthesized and post-translationally modified peptide; PKS, polyketide synthase

In order to gain a deeper insight into the genomic characteristics of *C. olla*, a comparative genomic analysis was conducted with three other *Cyathus* genomes that were available. Comparison of the composition of encoded genes showed that all four genomes had similar completeness, but *C. olla* SUT01 had the highest percentage of single-copy genes among them (Fig. 2A and Supplementary Table S8). Notably, both *Cyathus stercoreus* NK-82 and *Cyathus pallidus* NK-01 genomes had more than 60% duplicated genes (Fig. 2A), which implies that their genomes are not well assembled. Through homologous gene comparisons, a total of 7324 core genes were identified in the four genomes. Conversely, *C. stercoreus* NK-82 exhibited the highest abundance in GH, CBM, and PL types (Fig. 2C and Supplementary Table S9).

Furthermore, we analyzed the BGCs responsible for secondary metabolites, which are vital for the genome of medicinal mushrooms. Based on antiSMASH predictions, *C. stercoreus* NK-82 had the highest number of terpene BGCs, NRPS-like BGCs, and total BGCs, whereas *C. olla* SUT1 had the lowest (Fig. 2D and Supplementary Table S10). In terms of the number of CAZymes and BGCs, *C. stercoreus* NK-82 and *C. pallidus* NK-01 outperformed the other two species. These two species also possessed a higher number of unique genes compared to the others. This convergence is likely due to the poor assembly quality of the *C. stercoreus* NK-82 and *C. pallidus* NK-01 genomes (Table 1).

Despite their medicinal importance, *Cyathus* mushrooms have received less attention in genomic research than might be expected. Consequently, this study presents the inaugural comparative genomic analysis of *Cyathus* species based on the three available genomes. Comparative analyses of key functional genes and genomic features revealed variability and convergence in the genomes of *Cyathus* species.

### Phylogenetic and gene family variation analysis

**Fig. 3:**
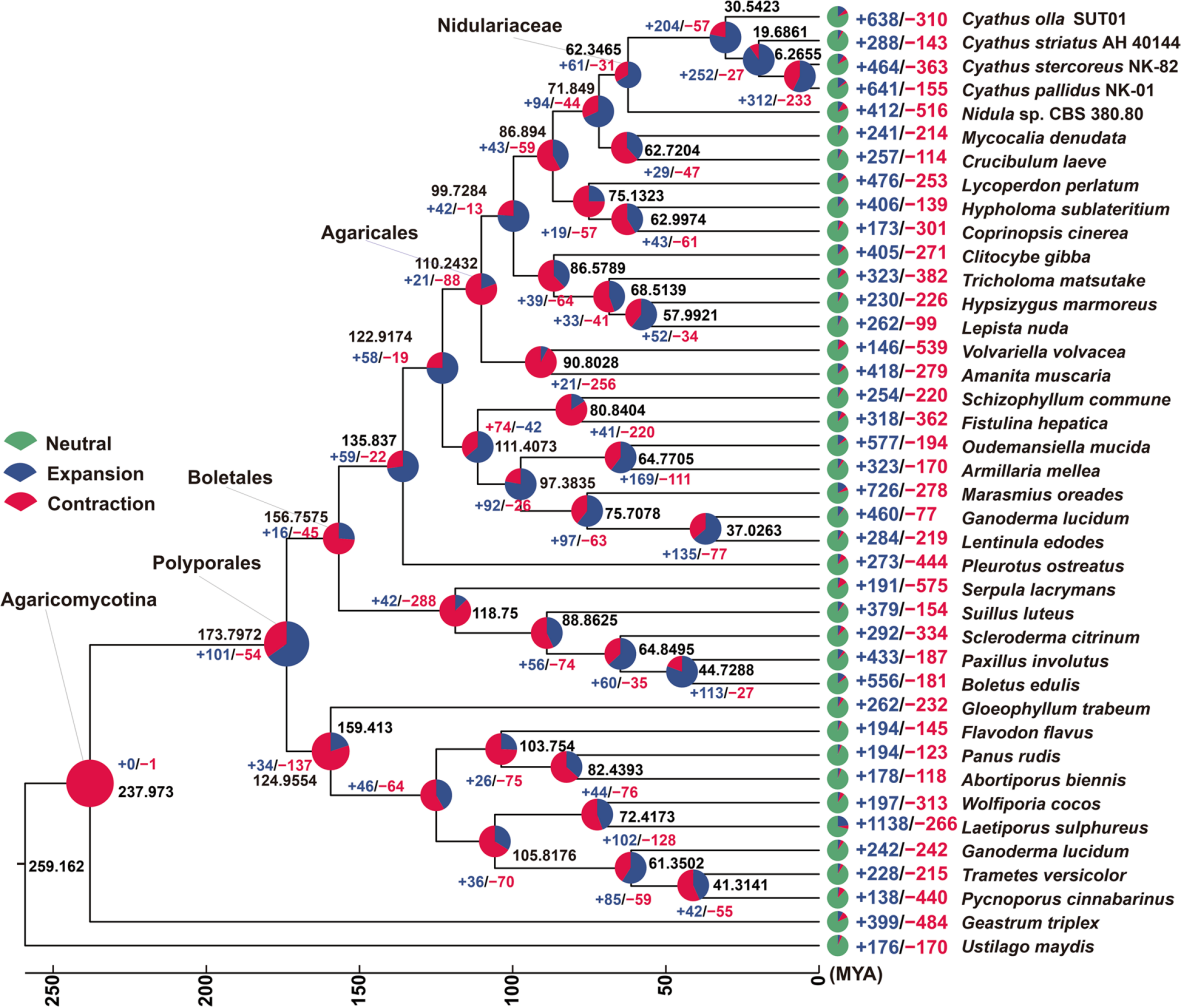
Phylogeny and gene family variation. The evolutionary relationship and expanded and contracted gene families among *Cyathus* species and 36 representative Basidiomycetes. The maximum likelihood method credibility tree was inferred from 40 single-copy orthologous genes. All nodes received full bootstrap support. The divergence time is labeled as the mean crown age for each node, while the 95% highest posterior density is also given within the *Cyathus* clade. The black numbers at the branches indicate the corresponding divergence times in MYA. The proportion of expansion and contraction in the genome of each species was displayed before its species name

To explore the potential evolutionary progression behind the increased biodiversity of *Cyathus* species, the divergence time of these species was estimated by constructing a phylogenomic tree that included four *Cyathus* species and 36 representative medicinal/edible mushrooms (Fig. 3). The phylogenomic tree inferred from an alignment of 236 single-copy orthologous genes from 633,550 genes delimited phylogenetic relationships among 40 species (Supplementary Table S11). The estimated average divergence time between Agaricomycotina and the outgroup, *Ustilago maydis*, is estimated to be 259.162 MYA with 95% highest posterior density (HPD) range of 120.53–325.28 MYA. The divergence times of the Agaricales order and its sister order are estimated to be 110.243 MYA (95% HPD of 88.36–228.59 MYA). The emergence of the Nidulariaceae family is estimated to have transpired approximately 62.347 MYA (95% HPD of 36.41 to 96.30 MYA) at the crown age. Within the Nidulariaceae family, the average divergence time between the genera *Nidula* and *Cyathus* is estimated to be 30.542 MYA (95% HPD of 31.52 to 84.14 MYA). Among the four species within the *Cyathus* genus, *C. olla* showed the earliest divergence, with an estimated time of 30.542 MYA (95% HPD of 15.14 to 43.39 MYA). Conversely, *C. pallidus* and *C. stercoreus* were found to be the most closely related, with a divergence time of 6.266 MYA (95% HPD of 3.04 to 9.32 MYA) (Fig. 3).

Further examination of the reconstructed evolutionary trees revealed intricate gene contraction and expansion events within the genomes of the 40 species, which encompassed a total of 633,550 genes. Within the *Cyathus* genus specifically, gene families in *C. olla*, *C. stratus*, *C. stercoreus*, and *C. pallidus* underwent varying degrees of expansion or contraction. Specifically, 638 gene families underwent expansion and 310 gene families experienced contraction in *C. olla*. In comparison, *C. stratus* had the least number of expansion events (288) and contraction events (143). *Cyathus stercoreus* had 464 expanded gene families and 363 contracted gene families, while *C. pallidus* had 641 expanded gene families and 155 contracted gene families (Fig. 3). It is reasonable to hypothesize that these gene family expansions and contractions have contributed to the phenotypic diversification and speciation processes within the *Cyathus* genus.

### TE analysis and genome duplication

A substantial proportion of the genome is comprised of repetitive sequences. A total of 26,177 repetitive sequences were predicted in the genome of *C. olla*, representing 22% of the entire genome and a total length of 10,737,448 base pairs. These repetitive sequences, also known as transposon elements (TEs), include short interspersed nuclear elements (SINEs), long interspersed nuclear elements (LINEs), long terminal repeat sequences (LTRs), and DNA transposon elements (DNA TEs). Among the four types of TEs, they represented 0.00% (10), 0.29% (569), 6.53% (3,137), and 1.23% (1,405) of the entire genome, respectively (Supplementary Table S12).

**Fig. 4:**
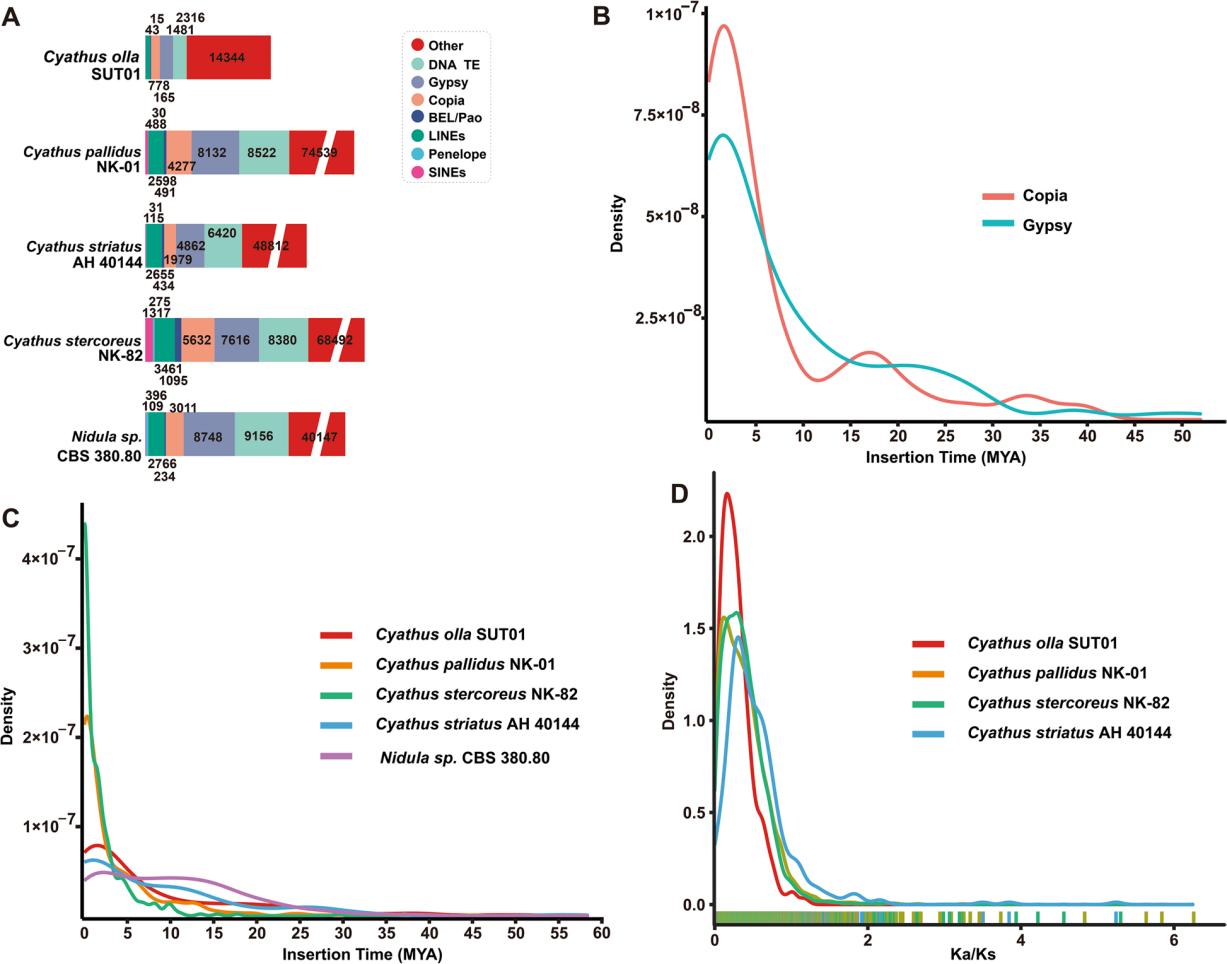
Analysis of TEs and positively selected genes in the *Cyathus olla* genome and foure closely related taxa. **A** Comparison of TE families in the five taxa. **B** Insertion bursts of Gypsy and Copia elements in *Cyathus olla* SUT01. **C** Comparison of temporal patterns of intact LTR-RT insertion bursts in the five taxa. **D** Frequency distributions of Ka/Ks between homologous gene pairs of the four taxa

The statistics indicate that the number and total length of repetitive sequences in *C. olla* and *C. striatus* were smaller than those in the other two species and smaller than those in the outgroup, *Nidula* sp. CBS 380.80. Cross-genome comparisons showed that other types of repetitive sequences contributed the most to the repetitive sequence expansion of these species, followed by DNA TEs. Additionally, *C. olla* had 2.83 Mb pairs of LTRs, including Gypsy, Copia, and unclassified LTR elements, which were the dominant TEs and occupied 5.18% of the genome (Fig. 4A). A continuous insertion of intact LTRs in *C. olla* has been observed since the end of the Quaternary glacial period, approximately two MYA, and it exhibited a distinct unimodal distribution (Fig. 4B).

Further analysis of TE insertions in the five species revealed that intact TEs were continuously inserted into the genomes of *C. olla*, *C. striatus,* and *Nidula* sp. Furthermore, the latest insertion times of intact TEs in the genomes of *C. stercoreus* and *C. pallidus* were nearly zero MYA (Fig. 4C). At the subtype level, the Gypsy and Copia-LTR elements in *C. olla* exhibited noticeable amplification peaks at approximately two MYA (Fig. 4C). Given the prevalence of Gypsy and Copia-LTRs in TEs, it is speculated that the most recent large-scale TE expansion in the genome of *C. olla* occurred around two MYA.

To gain insight into the impact of repetitive sequences on the genome, whole-genome duplication (WGD) analyses were conducted. The non-synonymous substitution rates (Ka), synonymous substitution rates (Ks), and their ratios (Ka/Ks) were calculated for homozygous gene pairs. Based on the distribution of Ks values of approximately 0.39 between orthologs, a WGD event was identified in *C. olla*, corresponding to divergences around 27.86 million years ago (Supplementary Fig. S9). In contrast, the Ks curves for the remaining three species peaked at lower values than that of *C. olla*, and their corresponding Ks values were all less than 0.39. The available evidence indicates that the WGD events occurred later and with less intensity in their genomes. These differences are further highlighted by the Ks/Ka curves (Fig. 4D).

### Bioactive secondary metabolites and their biosynthesis

**Fig. 5:**
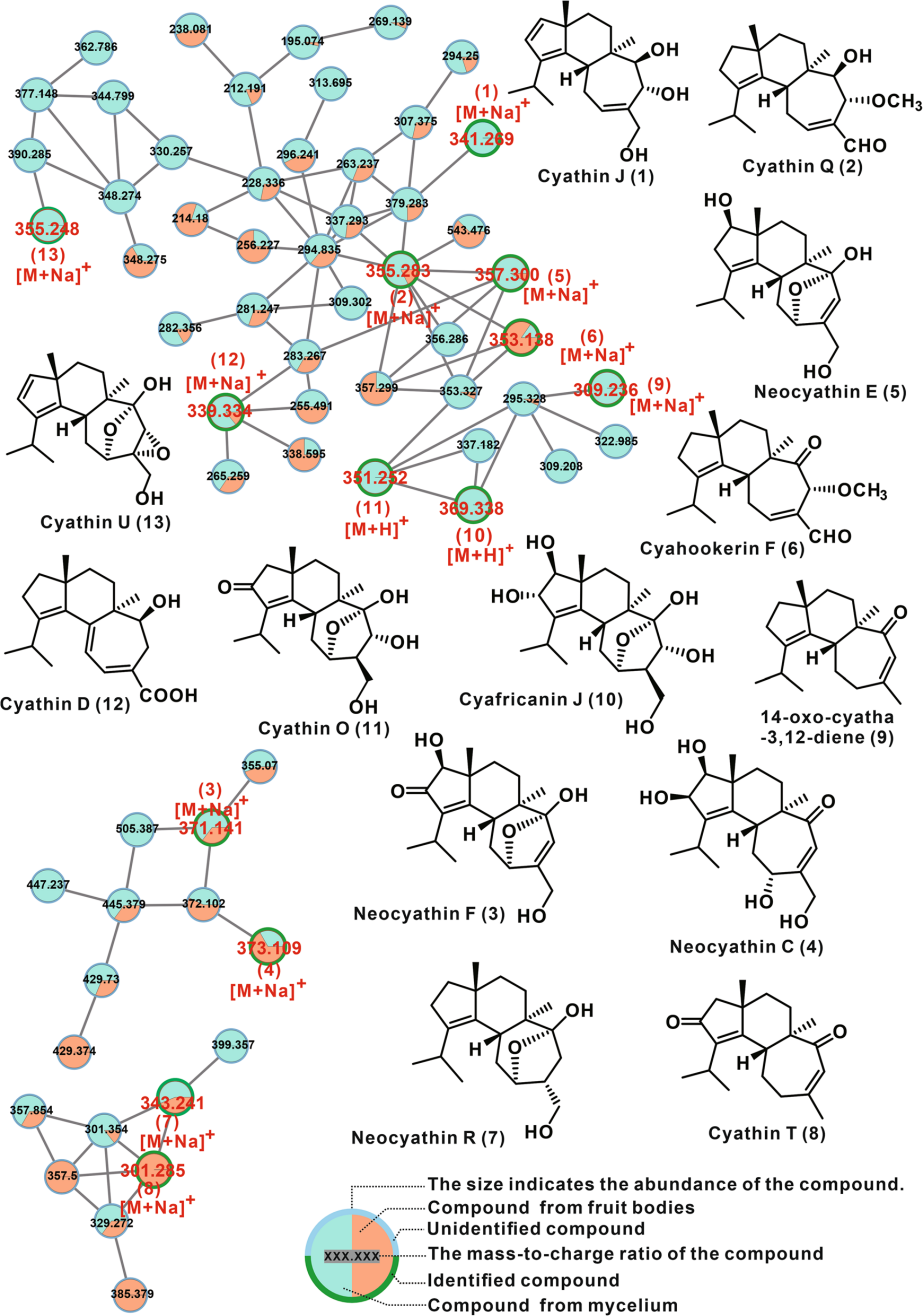
GNPS-based molecular network identification of metabolites from the fruiting bodies of *Cyathus olla* SUT01

Considering the potential application value of its anti-neurodegenerative and antimicrobial activities, the secondary metabolites of *C. olla* were investigated. The composition of the secondary metabolites in the fruiting body and PDB fermentation of the mycelia of C. olla did not exhibit any significant differences, as demonstrated by GNPS-based molecular networks (Fig. 5, and Supplementary Fig. S10). ESI-HRMS and NMR based analysis allowed the identification of 13 monomeric metabolites (Supplementary Fig. S11-14, Table 2, and Table S13). Structurally, all the 13 identified compounds are cyathane diterpenes. These identified cyathane diterpenes have shown anti-neurodegenerative activity in previous bioactivity investigations [22–24]. The bioactivity characterization of the crude extracts of the fruiting bodies of *C. olla* in the present study exhibited similar activity. This is the first time that the secondary metabolites of *C. olla* have been investigated.

**Table 2:** Identified metabolites from *C. olla* SUT01

No	Putative Metabolite	Molecular Formula	Adduct	m/z	Reference
**1**	cyathin J	C_20_H_30_O_3_	[M+Na]^+^	341.208	Wang *et. al* [22]
**2**	cyathin Q	C_21_H_32_O_3_	[M+Na]^+^	355.224	He *et. al* [14]
**3**	neocyathin F	C_20_H_28_O_5_	[M+Na]^+^	371.182	Wei *et. al* [23]
**4**	neocyathin C	C_20_H_30_O_5_	[M+Na]^+^	373.198	Wei *et. al* [23]
**5**	neocyathin E	C_20_H_30_O_4_	[M+Na]^+^	357.203	Wei *et. al* [23]
**6**	cyahookerin F	C_21_H_30_O_3_	[M+Na]^+^	353.208	Tang *et. al *[28]
**7**	neocyathin R	C_20_H_32_O_3_	[M+Na]^+^	343.224	Wei *et. al* [24]
**8**	cyathin T	C_20_H_28_O_2_	[M+H]^+^	301.216	Han *et. al *[29]
**9**	14-oxo-cyatha-3,12-diene	C_20_H_30_O	[M+Na]^+^	309.218	Tang *et. al *[28]
**10**	cyafricanin J	C_20_H_32_O_6_	[M+H]^+^	369.227	Yin *et. al *[30]
**11**	cyathin O	C_20_H_30_O_5_	[M+H]^+^	351.216	Wang *et. al* [22]
**12**	cyathin D	C_20_H_28_O_3_	[M+Na]^+^	339.193	Han *et. al *[31]
**13**	cyathin U	C_20_H_28_O_4_	[M+Na]^+^	355.188	Yu *et. al *[32]

**Fig. 6:**
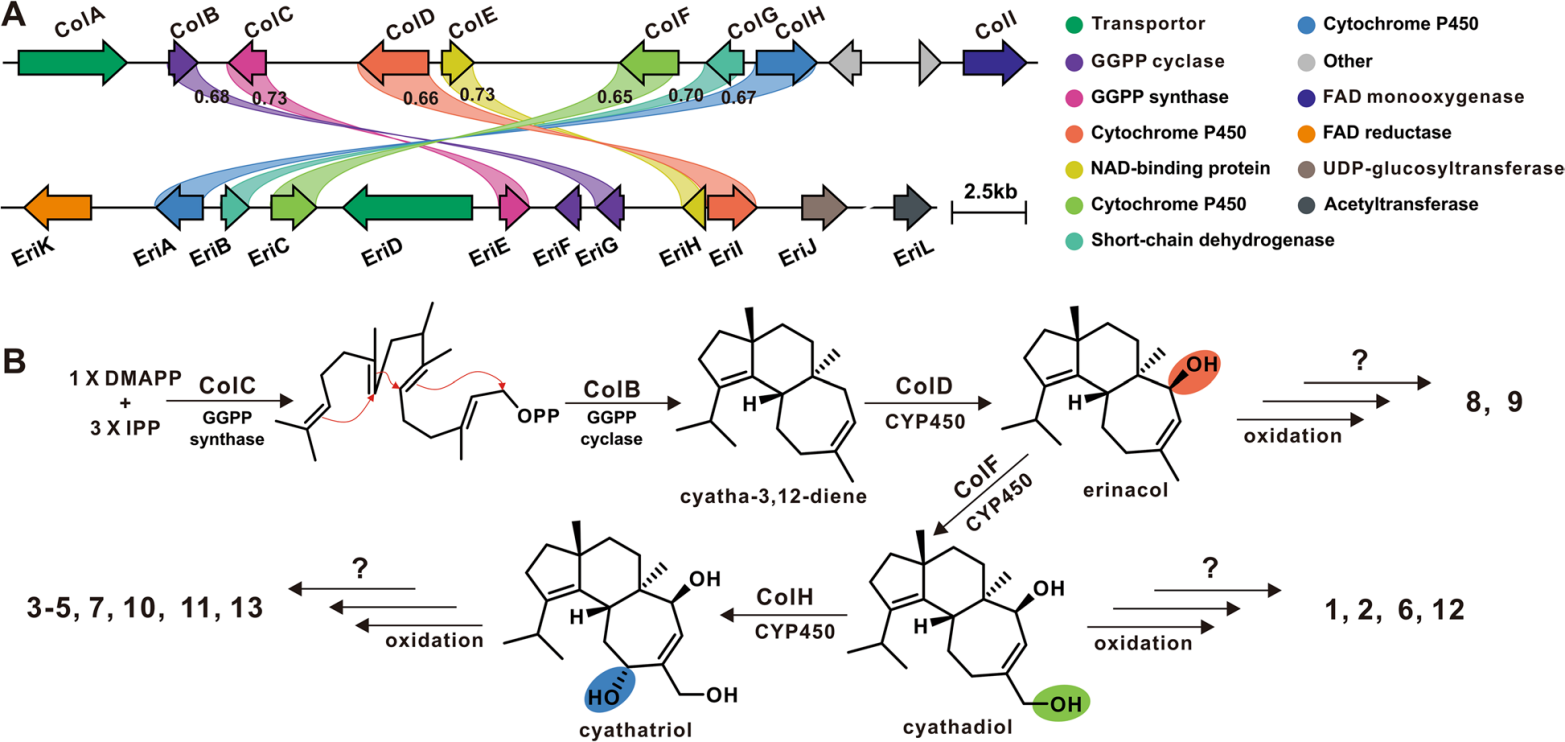
The proposed biosynthesis of cyathane diterpenoids of *Cyathus olla*. **A** Comparison of BGCs for erinacines of *Hericium* species and cyathane diterpenoids of *Cyathus olla*. **B** The proposed biosynthetic pathway of cyathane diterpenoids of *Cyathus olla*. GGPP indicates geranylgeranyl diphosphate

Erinacine-type cyathane diterpenes are representative diterpenoids derived from mushrooms, and their biosynthesis has been speculated [25, 26] and characterised [20, 27] in several species of *Hericium*. Given the conserved scaffold structure of cyathane diterpenes, we identified a candidate gene, g13493, on the genome of *C. olla* SUT1 using EriG as a probe. EriG [20, 27], the first identified cyathane diterpene cyclase from *H. erinaceus*, shares 67% amino acid sequence identity with g13493.t1. As a UbiA-type diterpene cyclase, EriG, distinguished from type I and type II diterpene cyclases, has two conserved motifs (NXXX(G/A)XXXD and DXXXD) ^21^. Multiple sequence alignment revealed that g13493.t1 contains both motifs (Supplementary Fig. S15), which suggests that it is a cyathane diterpene cyclase. Based on the position of g13493, we predicted the location of the BGC for the cyathane diterpene from *C. olla* SUT01, termed *Col* BGC (Fig 6A, Table 3). A total of six pairs of homologous genes were identified in both the Col BGC and the Eri BGC, exhibiting at least 65% identity at the amino acid sequence level. Of the nine functional genes in *Col* BGC, ColC is responsible for the synthesis of geranylgeranyl pyrophosphate (GGPP), while ColB catalysis the cyclization of GGPP to cyatha-3,12-diene, the backbone of the cyathane diterpene. Three cytochrome CP450 monooxygenases, ColD, ColF and ColH, mediate hydroxylation at C12, C14 methyl and C15 of the backbone. Based on the structural characteristics of the 13 identified metabolites, compounds **8** and **9** are hypothesized to be oxidized derivatives of the first-step hydroxylation product. On the other hand, compounds 1, 2, 6, and 12 are oxidation products of the second-step hydroxylation product and compounds 3-5, 7, 10, 11, and 13 are oxidation products of the third-step hydroxylation product. It is speculated that ColE, ColG and ColI are involved in these post-modification processes. UDP-glycosyltransferase, EriJ, is a key enzyme in the glycosylation modification of cyathane diterpenes in *H. erinaceus* [20], and xylosylated cyathane diterpenes tend to form complex polycyclic diterpenoids. However, no homologue of EriJ was found in the genome of *C. olla*, which suggests that *C. olla* does not produce glycosylated cyathane diterpenes. This is further corroborated by the results of its metabolite profiling.

**Table 3:** Predicted gene functions and blast results of the genes within *col* BGC

Gene	Accession numbers	Length (bp)	Predicted protein Length (aa)	BlastP homologs	Identities/Positives (%)	Predicted functions
*ColA*	WWA97550.1	3664	777	Q9FG72.1	26/44	Transporter
*ColB*	WWA97551.1	977	304	A0A1V0QSF1.1	70/83	Polyprenyl transferase EriG
*ColC*	WWA97552.1	1264	315	A0A1V0QSA8.1	73/84	Geranylgeranyl pyrophosphate synthase EriE
*ColD*	WWA97553.1	2337	516	A0A1V0QSE9.1	75/86	Cytochrome P450 monooxyhenase eriI
*ColE*	WWA97554.1	1088	250	A0A1V0QSC6.1	75/85	Short-chain dehydrogenase/reductase eriH
*ColF*	WWA97555.1	1983	518	A0A1V0QSB7.1	65/80	Cytochrome P450 monooxyhenase eriC
*ColG*	WWA97556.1	1263	313	A0A1V0QS34.1	71/83	Short-chain dehydrogenase/reductase eriB
*ColH*	WWA97557.1	2045	523	A0A1V0QSE7.1	71/84	Cytochrome P450 monooxyhenase eriA
*ColI*	WWA97558.1	2131	549	B1MM05.1	36/52	Flavin-dependent monooxygenase

### Biosynthetic potential mining

**Fig. 7:**
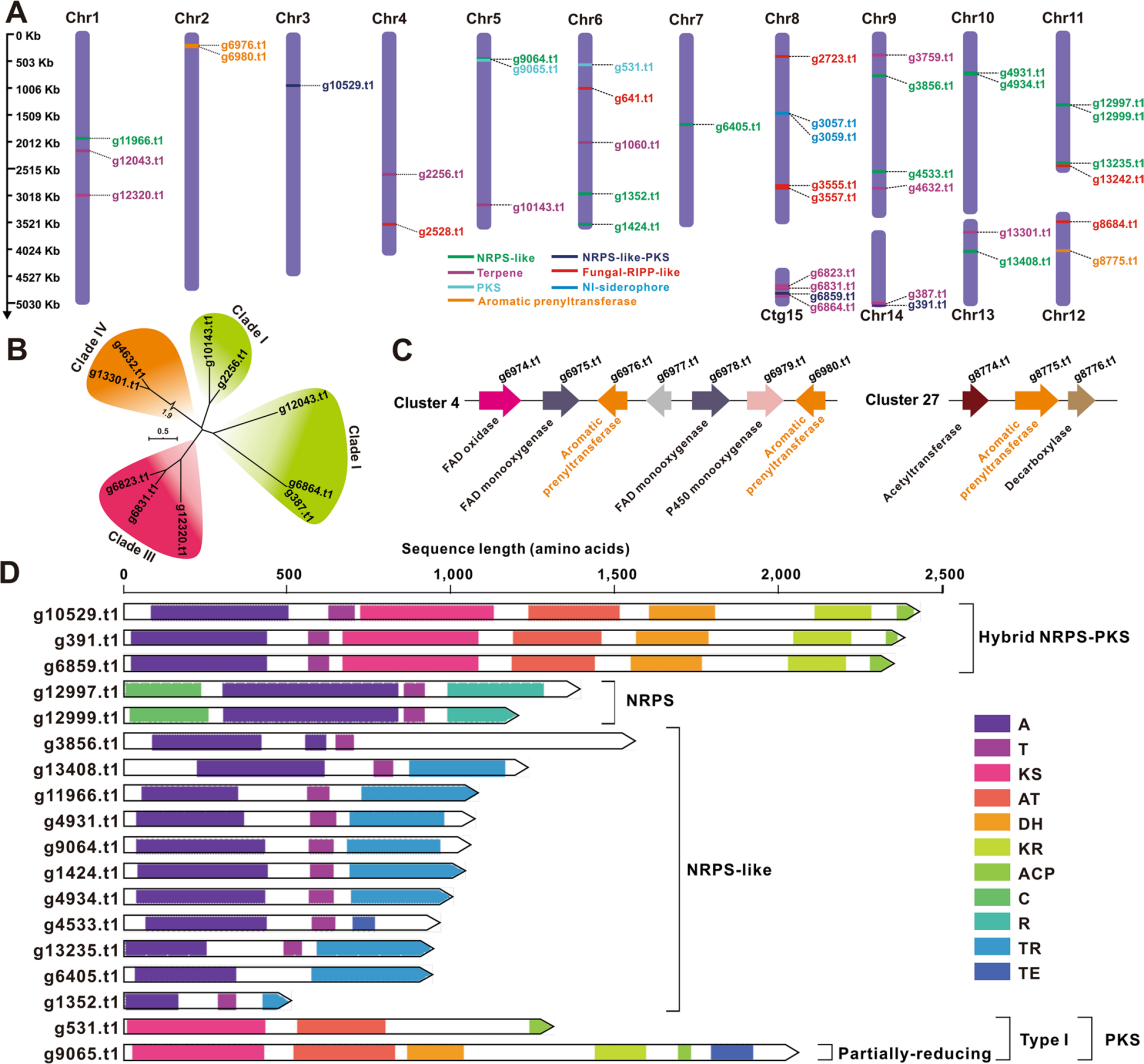
The core genes involved in secondary-metabolite biosynthesis from *Cyathus olla*. **A** Distribution of biosynthetic core genes for natural products on the chromosomes and contig, **B** Phylogenetic tree analysis for STSs, (**C**) Two putative BGCs containing aromatlc prenyltransferase. **D** Domain characterisation of the core enzymes containing multiple domains

Given the diverse range of medicinal compounds found in bird’s net fungi, research has been conducted to explore the biosynthetic potential of the C. olla species. The genome of *C. olla* SUT01 was predicted to contain 32 BGCs with 41 core genes using antiSMASH (Table 4). The core genes encode twelve terpenoid-related synthases, three aromatic prenyltransferases (PTs), sixteen NRPS-like enzymes, two PKSs, three NRPS-like-PKSs, six fungal-RiPP-likes, and two NI-siderophore enzymes. The aforementioned genes are distributed across fourteen chromosomes and a contig (Fig. 7A and Table 4).

**Table 4:** Putative BGCs responsible for the secondary metabolites of *C. olla*

Cluster No.	Location	start	end	core gene ID	core gene Type
1	Chr1	1,923,945	1,987,831	g11966.t1	NRPS-like
2	Chr1	2,186,798	2,202,549	g12043.t1	terpene
3	Chr1	3,004,730	3,035,804	g12320.t1	terpene
4	Chr2	202,765	249,038	g6976.t1,	aromatic prenyltransferase
				g6980.t1	aromatic prenyltransferase
5	Chr3	916,190	985,023	g10529.t1	NRPS-like-PKS
6	Chr4	2,598,151	2,629,349	g2256.t1	terpene
7	Chr4	3,509,967	3,602,251	g2528.t1	fungal-RiPP-like
8	Chr5	427,898	500,794	g9064.t1	NRPS-like
				g9065.t1	PKS
9	Chr5	3,183,041	3,216,197	g10143.t1	terpene
10	Chr6	541,425	605,744	g531.t1	PKS
11	Chr6	978,483	1,069,551	g641.t1	fungal-RiPP-like
12	Chr6	2,000,664	2,034,324	g1060.t1	terpene
13	Chr6	2,949,531	3,006,953	g1352.t1	NRPS-like
14	Chr6	3,540,348	3,604,359	g1424.t1	NRPS-like
15	Chr7	1,648,941	1,712,973	g6405.t1	NRPS-like
16	Chr8	402,631	438,992	g2723.t1	fungal-RiPP-like
17	Chr8	1,463,951	1,484,456	g3057.t1	NI-siderophore
				g3059.t1	NI-siderophore
18	Chr8	2,795,878	2,886,943	g3555.t1	fungal-RiPP-like
19	Chr9	375,107	404,055	g3759.t1	terpene
20	Chr9	751,217	810,116	g3856.t1	NRPS-like
21	Chr9	2,540,018	2,603,995	g4533.t1	NRPS-like
22	Chr9	2,861,166	2,891,497	g4632.t1	terpene
23	Chr10	723,905	802,129	g4931.t1	NRPS-like
				g4934.t1	NRPS-like
24	Chr11	1,320,012	1,385,244	g12997.t1	NRPS-like
				g12999.t1	NRPS-like
25	Chr11	2,405,979	2,553,849	g13235.t1	NRPS-like
				g13242.t1	RiPP-like
26	Chr12	131,654	225,210	g8684.t1	RiPP-like
27	Chr12	706,295	738,190	g8775.t1	aromatic prenyltransferase
28	Chr13	184,249	215,218	g13301.t1	terpene
29	Chr13	530,717	595,235	g13408.t1	NRPS-like
30	Chr 14	1,341,120	1,387,289	g387.t1	terpene
				g391.t1	NRPS-like-PKS
31	Ctg15	302,784	350,184	g6823.t1	terpene
				g6831.t1	terpene
32	Ctg15	403,441	471,790	g6859.t1	NRPS-like-PKS
				g6864.t1	terpene

Among the twelve terpenoid-related enzymes (Supplementary Table S14), ten of them are sesquiterpene synthases (STSs). Cluster analysis of the STSs from *C. olla* and identified STSs from Basidiomycota revealed that the ten STSs belong to Clade I, II, and IV, with no members belonging to Clade III (Fig. 7Band Supplementary Fig. S16). Previous studies on natural product chemistry have identified various sesquiterpenes, such as illudalane, bisabolane, drimane, protoilludane, eudesmane, and others, in the metabolites of the genus *Cyathus* [13]. It is speculated that these nine STSs are responsible for the production of these sesquiterpenes. Additionally, ten enzymes associated with triterpene synthesis, including presqualene diphosphate synthase (g1060.t1) and squalene synthase (g3759.t1.t1), were identified through sequence similarity alignment (Supplementary Table S14) and annotated by KEGG (Supplementary Fig. S17). Furthermore, three aromatic PTs showed approximately 30% identity with tyrosine *O*-prenyltransferase (Supplementary Table S14). They potentially form two BGCs with common natural product biosynthetic post-modification genes, such as FAD monooxygenase and Cytochrome C P450 (Fig. 7C).

A comparison was conducted between 21 core enzymes with multiple domains. Among them, three hybrid NRPS-like-PKSs were observed to share the same A-T-KS-AT-DH-KR-ACP domains. Two NRPS-like enzymes, 12999.t1 and 12997.t1, were defined as NRPS-like by antiSMASH, but labelled as NRPS by Synthaser due to the presence of the C domain at their N-terminus. All eight NRPS-like enzymes, including g13408.t1, possess A-T-TR domains. Another NRPS-like enzyme, g4533.t1, contains A-T-TE domains. The remaining two NRPS-like enzymes, g3856.t1 and g6405.t1, only had two domains, with the former being A-T and the latter being A-TE. Two type I PKSs, g531.t1 and g9065.t1, were observed to contain KS-AT-ACP and KS-AT-DH-KR-ACP-TE domains, respectively. The latter was identified as a partially reducing PKS (Fig. 7D, Supplementary Table S15). Previous studies have identified several polyketides and cyclic dipeptides in the secondary metabolites of the genus *Cyathus* [13]. It is proposed that the biosynthesis of these compounds is correlated with these multi-domain enzymes. Additionally, the genome of *C. olla* contains seven genes that putatively encode RIPP-like enzymes, which have not yet been deciphered in Basidiomycota. Among them, six genes encode products that are most similar to the phomopsin biosynthesis cluster protein Yc, sharing around 30% identity (Supplementary Table S16). Furthermore, the genome also contains two Ni-siderophores that do not rely on NRPS (Supplementary Table S16). The presence of these diverse core genes indicates that *C. olla is capable of* to producing a wide range of secondary metabolites.

## Conclusions

This study presents a high-quality chromosome-level genome assembly of *C. olla*, comprising fourteen pseudochromosomes with a total length of 47.83 Mb, containing 14,248 protein-coding genes, 205 tRNAs, 27 snRNAs, 10 rRNAs and one sRNA. Comparative genomic analyses have revealed the core genes of the genus *Cyathus* and the unique genes of four species. Specific functional gene comparisons reveal quantitative differences between the four species in genes encoding CAZymes and BGCs. A phylogenetic analysis of 236 single-copy homologous genes revealed that *C. olla* diverged earliest within the genus *Cyathus*, with a divergence age of 30.54 Mya. With regard to genome duplication, the LTR-RT and Ka/Ks analysis indicates that the *C. olla* genome has undergone apparent WGD events, which implies that the increase in LTR-RT insertions and tandem gene duplications within the *C. olla* genome may have contributed to its genome variation and adaptation to the external environment. Secondary metabolite investigation has validated the possible medicinally active constituents, cyathane diterpenes, of *C. olla*. Genome mining has identified the BGC for cyathane diterpenes in *C. olla* and speculated on their biosynthetic pathway. Additionally, 32 BGCs with 41 core genes have been predicted and discovered from the genome of *C. olla*. Moreover, this high-quality reference genome will accelerate the study of the biosynthesis of medicinal compounds in *Cyathus,* and provide important genetic resources for molecular breeding in medicinal *Cyathus* mushrooms. In conclusion, this presentation provides an excellent resource for further research into the genetics and biochemistry of *C. olla* and its potential medicinal applications.

### Supplementary Information

The online version contains supplementary material available at https://doi. org/XXXXXX.

Supplementary Material 1. 

## Data Availability

The ITS sequence of *C. olla* SUT01 was registered in the NCBI GenBank under accession number PP376222 and the final genome assembly results and related data were submitted to NCBI under BioProject PRJNA1080487, BioSample SAMN40136088, and SAR SRR28092570, SRR28106886, and SRR28106977 respectively. The BGC (Col BGC) for cyathane diterpene from *C. olla* SUT01 was deposited in the NCBI GenBank under accession number PP341330. The network file based on positive-ion mode MS data can be found and accessed at https://gnps.ucsd.edu/ProteoSAFe/status.jsp?task=f7e820d3c3634a3a834c427ecd4fb06a. The NMR data of compounds 3-5 have been submitted to NP-MRD (Deposition ID: NPd000000628), and are available to download at this link: https://depositions.np-mrd.org/request-data/844da2cd-623a-4a21-a946-692136eccf12.
