# Supplementary material for: Genome-wide characterization and metabolite profiling of Cyathus olla: insights into the biosynthesis of medicinal compounds
Source: BMC Genomics. 2024 Jun 19;25:618. doi: 10.1186/s12864-024-10528-3 (PMC11186289; doi:10.1186/s12864-024-10528-3)
Supplement: Supplementary file 1 — Supplementary Material 1. [file 12864_2024_10528_MOESM1_ESM.pdf]

Genome-Wide Characterization and Metabolite Profiling of *Cyathus olla*: Insights into the Biosynthesis of Medicinal Compounds

Xiuchao Xie[1], Ling Zhao[2], Yu Song[1], Yanming Qiao[1], Zhen-Xin Wang[3], Jianzhao Qi[1, 3, *]

[1]Shaanxi Province Key Laboratory of Bio-resources, Qinba State Key Laboratory of Biological Resources and Ecological Environment (Incubation), School of Biological Science and Engineering, Shaanxi University of Technology, Hanzhong 723000, China

[2]Department of Pharmacy, School of Medicine, Xi'an International University, Xi'an 710077, China.

[3]Shaanxi Key Laboratory of Natural Products & Chemical Biology, College of Chemistry & Pharmacy, Northwest A&F University, Yangling 712100, China.

*To whom correspondence should be addressed. Tel: +029-87092381. Email: qjz@nwafu.edu.cn

Content

Experimental section: Determination of neuroprotective activity of crude extract of *C. olla* ..1
Table S1. Statistics of Illumina NovaSeq sequencing data volume information of *C. olla* SUT01 genome. 2
Table S2. Statistics of Oxford Nanopore PromethION sequencing data volume of *C. olla* SUT01 genome. 3
Table S3. Statistics on genome assembly of *C. olla* SUT01. 4
Table S4. Statistics of Illumina NovaSeq sequencing data mapping of *C. olla* SUT01 genome. 5
Table S5. Annotation Statistics of coding gene from the *C.olla* SUT01 genome. 6
Table S6. Statistics of non-coding RNA annotation results in *C.olla* SUT01 genome. 7
Table S7. Statistics of *C. olla* SUT01 protein-coding gene annotation. 8
Table S8. Comparison of genome assembly quality based on BUSCO. 9
Table S9. Comparison of CAZyme-related gene families of four *Cyathus* species 10
Table S10. The core gene for secondary metabolite biosynthesis in four *Cyathus* species.....11
Table S11. The source (URL) statistics for 39 Basidiomycetes and *C. olla* used to phylogenetic analysis. 12
Table S12. Statistics of repetitive sequence of *C. olla* SUT01 13
Table S13. The NMR data of compounds from *C. olla* SUT01. 14
Table S14. Terpenoid biosynthesis related enzymes in *C. olla* SUT01 genome. 15
Table S15. Core genes with multi-domain in *C. olla* SUT01 genome. 16
Table S16. RIPP-like and NI-siderophore enzymes in *C. olla* SUT01 genome. 17
Figure S1. The ITS of *Cyathus olla* SUT01 was aligned to NCBI nr database. 18
Figure S2. NGF-dependent promotional activity of crude extract of *C. olla* on rat pheochromocytoma PC12 cells. 19
Figure S3. Kmer-Depth and Kmer Species-Frequency Distribution Plot. 20
Figure S4. Species distribution map of Nr database alignment to sequences. 21
Figure S5. Statistical map of functional annotation classification based on GO database......22
Figure S6. Statistical Chart of COG Functional Annotated Classification. 23
Figure S7. KEGG Pathway Functional Classification Chart. 24
Figure S8. Domain annotation based on the Pfam database. 25
Figure S9. Ka and Ks comparisons of four *Cyathus* species 26
Figure S10. Molecular network analysis of metabolites from the mycelium and fruiting bodies of *C. olla* SUT01. 27
Figure S11. The LC-ESI-HRMS and LC-ESI-HRMS/MS spectrums of isolates from *C. olla* SUT01. 28
Figure S12. The ^{1}H and ^{13}C NMR spectra of Compound **3** 29
Figure S13. The ^{1}H and ^{13}C NMR spectra of Compound **4** 30
Figure S14. The ^{1}H and ^{13}C NMR spectra of Compound **5** 31
Figure S15. The two conserved motifs in proposed cyathane diterpene cyclase from *C. olla* SUT01. 32
Figure S16. FPPs Cluster analysis of the strain *C. olla* SUT01 and other Basidiomycetes. ...33
Figure S17. Annotation of the triterpenoid biosynthetic pathway of *C. olla* SUT01 using KAAS. 34
Reference 35

Experimental section: Determination of neuroprotective activity of crude extract of *C. olla*

The rat adrenal pheochromocytoma cell line PC-12 was obtained from the China Center for Typical Culture Collection. The cells were cultured in F-12 (Ham) nutrient mixture supplemented with 10% heat-inactivated horse serum (HS), 5% heat-inactivated fetal bovine serum (FBS), 100 U/mL penicillin G, 100 g/mL streptomycin, and 2.5 g/L sodium bicarbonate. The PC-12 cells were cultured in a humidified atmosphere with 5% CO_2 at 37°C.

PC-12 cells with neurites were morphologically analyzed and quantified using phase contrast microscopy. The cells were inoculated onto poly-L-lysine-coated 24-well plates at a density of 2×10^4 cells/mL in standard serum medium and incubated for 24 hours. Prior to exposure to either the vehicle (0.1% DMSO) or the specified reagents, the F-12 medium with low serum (1% HS and 0.5% FBS) was replaced. The cells were then treated with 20 ng/mL of nerve growth factor (NGF) and varying concentrations of crude extract of *C. olla*. A control group was also treated with 20 ng/mL NGF. This experiment was repeated three times, with one concentration in each well. After an additional 48 hours of incubation, the neuronal growth of PC-12 cells was captured using a digital camera attached to an inverted microscope equipped with a phase contrast objective. Five random images were chosen from each well under the microscope. The proportion of cells with neurites equal to or longer than the length of one cell body was determined as a measure of neurite outgrowth, expressed as a percentage of the total number of cells within the five randomly selected fields of view. The experiment was repeated at least three times, and the results are presented as the mean ± standard deviation.

Table S1. Statistics of Illumina NovaSeq sequencing data volume information of *C. olla* SUT01 genome.

Sample_name	Total_reads	Total_bases	GC_content	Q20	Q30
rawdata	40,012,428	6,001,864,200	43.80%	97.69%	93.29%
cleandata	40,012,222	6,001,833,300	43.80%	97.69%	93.29%

Sample name is data type. **Total_reads** is sequencing reads number. **Total_bases** is total sequencing base number. **GC_Content** is G/C base number as a percentage of total base number. **Q20** and **Q30** are Phred value greater than 20, 30 base as a percentage of total base, respectively.

Table S2. Statistics of Oxford Nanopore PromethION sequencing data volume of *C. olla* SUT01 genome.

Rank	Flag	TotalBase	TotalReads	MaxLen	AvgLen	N50	L50	N90	L90	meanQ
>0	all	6,414,667,997	809,754	131,441	7,921.74	17,224	114,910	3,251	450,470	11.07
>0	pass	6,152,930,142	774,024	131,441	7,949.27	17,259	110,134	3,262	430,771	11.30
>0	fail	261,737,855	35,730	98,603	7,325.43	16,437	4,784	3,003	19,744	6.14
>5000	all	5,307,930,082	334,581	131,441	15,864.4	20,814	85,711	7,666	248,683	11.10
>5000	pass	5,096,046,542	320,984	131,441	15,876.32	20,827	82,283	7,675	238,562	11.31
>5000	fail	211,883,540	13,597	98,603	15,583.1	20,516	3,429	7,475	10,125	6.15
>10000	all	4,378,770,529	203,261	131,441	21,542.6	24,020	64,930	12,656	164,424	11.10
>10000	pass	4,206,400,251	195,256	131,441	21,543	24,030	62,396	12,659	157,955	11.30
>10000	fail	172,370,278	8,005	98,603	21,532.82	23,808	2,535	12,627	6,470	6.16
>50000	all	212,418,872	3,663	131,441	57,990.41	56,315	1,651	51,057	3,243	10.76
>50000	pass	202,439,365	3,493	131,441	57,955.73	56,286	1,576	51,076	3,093	10.98
>50000	fail	9,979,507	170	98,603	58,702.98	56,760	76	50,863	151	6.19

Rank is the gradient of data length, **>0** is that all data. **Flag** is data type, all is all sequencing data, pass is effective sequencing data, **fail** is filtration data. **TotalBase** is the number of base. **TotalReads** is the number of reads. **MaxLen** is maximum length of data. **AvgLen** is average length of data. **N50** is N50 of data, all reads are summed in order from long to short, and when additive length up to half of all reads total length, the last read length added is N50. **L50** is L50 of data, all reads are accumulated in turn ranking in order from long to short, when additive length up to half of all reads total length, the number of sequences is L50. **N90** is N90 of data, algorithm the same as N50. **L90** is L90 of data, algorithm the same asL50. **meanQ** is mean quality value.

Table S3. Statistics on genome assembly of *C. olla* SUT01.

Item	Value
Total_length(bp)	47,825,376
Total_length_without N(bp)	47,825,376
Total_number	15
GC_content (%)	43.73
N50(bp)	3,637,865
N90(bp)	1,729,561
Average(bp)	3,188,358.40
Median(bp)	3,524,905.00
Min(bp)	656,961
Max(bp)	5,034,100

Total_length is the assembly length. **Total_length_without N** is the length without gap in the assembly result. **Total_number** is the number of assembled sequences. **GC_content** is the GC content. **N50** is the N50 of the data, all the sequences are sorted according to the longest to the shortest and then added up, and the last added sequence length is N50 when it reaches half of the total length. **N90** algorithm is the same as N50. **Average** is the average length. **Median** is the median length. **Min** is the minimum length. **Max** is the maximum length.

Table S4. Statistics of Illumina NovaSeq sequencing data mapping of *C. olla* SUT01 genome.

Type	Number
map_rate	90.03%
Average_depth	106.55
Coverage	99.91%

map_rate is Illumina NovaSeq sequencing data mapping rates. **Average_depth** is average coverage depth. **Coverage** is coverage rate.

Table S5. Annotation Statistics of coding gene from the *C.olla* SUT01 genome.

Type	Number
Total number of gene	14,248
Average of mRNA length	1,548.17
Average of cds length	1,120.64
Average of exon number	6.73
Average of exon length	166.55
Average of intron length	74.62
Total number of exon	95,870
Total number of intron	81,622
Total intron length	6,090,354

The total number of gene is the total number of genes; the average of mRNA_length is the average length of mRNA; the average of cds_length is the average length of CDS; the average of exon_number is the average number of exons per gene. the average of exon_length is the average exon length; the average of intron_length is the average intron length; the total number of exon is the total number of exons; the total number of intron is the total number of introns; the total intron length is the total intron length.

Table S6. Statistics of non-coding RNA annotation results in *C.olla* SUT01 genome.

Class	number	totalLen(bp)	meanLen(bp)
rRNA	10	8,445	844
sRNA	1	345	345
snRNA	27	3,600	133
tRNA	205	18,503	90

rRNA is ribosomal RNA; tRNA is transport RNA; sRNA is small regulatory RNA; snRNA is nucleolar small RNA. **totalLen** and **meanLen** are the total length and mean length.

Table S7. Statistics of *C. olla* SUT01 protein-coding gene annotation.

Item	Count	Percentage
All	14,248	100%
Annotation	11,966	83.98%
Uniprot	6,265	43.97%
Pfam	8,867	62.23%
Refseq	4,532	31.81%
Nr	11,721	82.26%
Interproscan	8,938	62.73%
GO	6,216	43.63%
KEGG	4,551	31.94%
Pathway	2,788	19.57%
COG	1,261	8.85%

Annotation is the gene with at least one annotation; Uniprot is the gene annotated to the Uniprot database; Pfam is the gene that is annotated to the Pfam database; Refseq is the gene that is annotated to the Refseq database; Nr is the gene that is annotated to the Nr database; Interproscan is the gene that is annotated to the Interproscan GO is the gene annotated to the GO database; KEGG is the gene that is annotated to the KEGG database; Pathway is the gene that is annotated to the KEGG Pathway database; COG is the gene that is annotated to the COG database.

Table S8. Comparison of genome assembly quality based on BUSCO.

Species	Completed (%)	Single-copy (%)	Duplicated (%)	Fragmented (%)	Missing (%)
Cyathus olla SUT01	97.89	96.44	1.45	1.58	0.53
Cyathus pallidus NK-01	98.55	38.13	60.42	1.32	0.13
Cyathus stercoreus NK-82	98.95	7.26	91.69	1.06	0
Cyathus striatus AH 40144	97.23	87.2	10.03	1.85	0.92

Table S9. Comparison of CAZyme-related gene families of four *Cyathus* species

Species	GH	AA	CBM	CE	GT	PL
***Cyathus olla* SUT01**	255	128	13	28	72	19
***Cyathus striatus* AH 40144**	272	191	20	34	84	19
***Cyathus pallidus* NK-01**	400	306	86	59	103	26
***Cyathus stercoreus* NK-82**	418	302	122	43	95	28

Table S10. The core gene for secondary metabolite biosynthesis in four *Cyathus* species.

	Cyathus olla **SUT01**	***Cyathus striatus*** **AH 40144**	***Cyathus stercoreus*** **NK-82**	***Cyathus pallidus*** **NK-01**
**Total**	41	82	202	209
**Terpene**	12	31	84	95
**NRPS-like**	13	18	39	40
**Fungal-RiPP-like**	6	25	57	52
**PKS**	2	1	9	6
**Aromatic prenyltransferase**	3	2	11	12
**NI-siderophore**	2	2	1	1
**NRPS-like-PKS**	3	2	0	2
**NRPS**	0	1	1	1

Table S11. The source (URL) statistics for 39 Basidiomycetes and *C. olla* used to phylogenetic analysis.

Species	Source
Cyathus olla SUT01	in the study
Cyathus stercoreus NK-82	https://genome.jgi.doe.gov/portal/Cyaste1/Cyaste1.download.html
Cyathus pallidus NK-01	https://genome.jgi.doe.gov/portal/Cyapal1/Cyapal1.download.html
Cyathus striatus AH 40144	https://genome.jgi.doe.gov/portal/Cyastr2/Cyastr2.download.html
Nidula sp. CBS 380.80	https://genome.jgi.doe.gov/portal/Nidsp1/Nidsp1.download.html
Mycocalia denudata	https://genome.jgi.doe.gov/portal/Mycden1/Mycden1.download.html
Crucibulum laeve	https://genome.jgi.doe.gov/portal/Crula1/Crula1.download.html
Lycoperdon perlatum	https://genome.jgi.doe.gov/portal/Lycper1/Lycper1.download.html
Hypholoma sublateritium	https://genome.jgi.doe.gov/portal/Hypsu1/Hypsu1.download.html
Coprinopsis cinerea	https://www.ncbi.nlm.nih.gov/datasets/genome/GCF_000182895.1/
Clitocybe gibba	https://genome.jgi.doe.gov/portal/Cligib1/Cligib1.download.html
Tricholoma matsutake	https://genome.jgi.doe.gov/portal/Trima3/Trima3.download.html
Hypsizygus marmoreus	https://genome.jgi.doe.gov/portal/Hypma1/Hypma1.download.html
Lepista nuda	https://genome.jgi.doe.gov/portal/Lepnud1/Lepnud1.download.html
Volvariella volvacea	https://genome.jgi.doe.gov/portal/Volvo1/Volvo1.download.html
Amanita muscaria	https://genome.jgi.doe.gov/portal/Amamu1/Amamu1.download.html
Schizophyllum commune	https://genome.jgi.doe.gov/portal/SchcoZB1/SchcoZB1.download.html
Fistulina hepatica	https://genome.jgi.doe.gov/portal/Fishe1/Fishe1.download.html
Oudemansiella mucida	https://genome.jgi.doe.gov/portal/Oudmuc1/Oudmuc1.download.html
Armillaria mellea	https://genome.jgi.doe.gov/portal/Armme1_1/Armme1_1.download.html
Marasmius oreades	https://www.ncbi.nlm.nih.gov/datasets/genome/GCF_018924745.1/
Ganoderma lucidum	https://genome.jgi.doe.gov/portal/Ganluc1/Ganluc1.download.html
Lentinula edodes	https://genome.jgi.doe.gov/portal/Led_CS584_1/Led_CS584_1.download.html
Pleurotus ostreatus	https://genome.jgi.doe.gov/portal/PleosPC15_2/PleosPC15_2.download.html
Serpula lacrymans	https://www.ncbi.nlm.nih.gov/datasets/genome/GCF_000218685.1/
Suillus luteus	https://www.ncbi.nlm.nih.gov/datasets/genome/GCA_000827255.1/
Scleroderma citrinum	https://genome.jgi.doe.gov/portal/Sclcihr1/Sclcihr1.download.html
Paxillus involutus	https://genome.jgi.doe.gov/portal/Paxin1/Paxin1.download.html
Boletus edulis	https://genome.jgi.doe.gov/portal/Boledp1/Boledp1.download.html
Gloeophyllum trabeum	https://genome.jgi.doe.gov/portal/Glotr1_1/Glotr1_1.download.html
Flavodon flavus	https://genome.jgi.doe.gov/portal/Flafl1/Flafl1.download.html
Panus rudis	https://genome.jgi.doe.gov/portal/Panru1/Panru1.download.html
Abortiporus biennis	https://genome.jgi.doe.gov/portal/Abobie1/Abobie1.download.html
Wolfiporia cocos	https://genome.jgi.doe.gov/portal/Wolco1/Wolco1.download.html
Laetiporus sulphureus	https://genome.jgi.doe.gov/portal/Laesu1/Laesu1.download.html
Ganoderma lucidum	https://genome.jgi.doe.gov/portal/Ganluc1/Ganluc1.download.html
Trametes versicolor	https://genome.jgi.doe.gov/portal/Trave1/Trave1.download.html
Pycnoporus cinnabarinus	https://genome.jgi.doe.gov/portal/Pycci1/Pycci1.download.html
Geastrum triplex	https://genome.jgi.doe.gov/portal/Geatri1/Geatri1.download.html
Ustilago maydis	https://genome.jgi.doe.gov/portal/Ustma2/Ustma2_2.download.html

Table S12. Statistics of repetitive sequence of *C. olla* SUT01.

Item	Subfamily	Number	Length(bp)	Coverage
SINE	/	10	764	0.00%
LINE	/	569	139,509	0.29%
LTR	/	3,137	3,124,044	6.53%
LTR	Gypsy	1,998	2,405,457	5.03%
LTR	Copia	954	705,874	1.48%
DNA	/	1,405	587,157	1.23%
Satellite	/	24	2,049	0.00%
Simple_repeat	/	4,159	180,299	0.38%
Low_complexity	/	1,098	53,042	0.11%
Other	/	222	101,090	0.21%
Unknown	/	15,553	6,674,771	13.96%
Total	/	26,177	10,737,478	22.45%

SINE is short scattered element. **LINE** is Long scattered element. **LTR** is long terminal repetition, mainly include two types, Gypsy and Copia. **DNA** is transposons. **Satellite** is satellite repetitive sequence. **Low_complexity** is Low_complexity repetition. **Other** is other types repetition. **Unknown** is unknown repetitive sequence. **Total** is total repetitive sequence.

Table S13. The NMR data of compounds from *C. olla* SUT01.

Position	Neocyathin F (**3**)		Neocyathin C (**4**)		Neocyathin E (**5**)	
	δ_C	δ_H (mult., *J* in Hz)	δ_C	δ_H (mult., *J* in Hz)	δ_C	δ_H (mult., *J* in Hz)
1	84.5	3.80 s	88.6	3.67 d (5.4)	82.9	3.83 dd (9.7, 8.0)
2	209.5		83.6	4.54 d (5.6)	37.3	2.48 dd (14.6, 7.8)
						2.13 m
3	141.6		140.2		137.2	
4	174.1		141.5		137.8	
5	43.3	3.06 dd (12.7, 4.2)	39.1	2.72 d (8.5)	41.6	2.54 m
6	44.9		56.6		42.7	
7	30.5	1.71 m	34.9	1.71 m	31.1	1.59 m
		1.57 m		1.41 m		1.38 m
8	35.3	1.91 m	30.3	1.56 m	36.3	1.69 m
		1.78 m		1.38 m		1.51 td (13.5, 4.5)
9	47.3		48.7		50.0	
10	27.6	2.42 td (12.7,3.6)	37.1	2.51 m	28.3	2.22 m
		1.68 m		1.92 m		1.62 m
11	79.9	4.78 m	72.5	4.18 m	80.1	4.73 m
12	149.3		157.1		149.1	
13	126.9	6.12 s	123.2	6.12 s	126.7	6.03 s
14	110.8		210.7		111.3	
15	58.9	4.29 d (15.4)	64.6	4.37 dd (16.1)	58.9	4.26 m
		4.24 d (14.1)		4.26 d (15.7)		
16	12.3	1.09 s	15.7	1.09 s	12.1	1.02 s
17	22.4	1.13 s	23.7	1.13 s	17.4	0.95 s
18	26.3	3.14 p (7.0)	28.3	2.85 p (6.9)	27.5	2.98 m
19	20.3	1.22 d (7.0)	24.4	1.22 d (7.0)	21.3	0.93 d (5.5)
20	21.3	1.21 d (6.7)	19.6	1.15 d (7.0)	22.6	1.05 d (6.7)

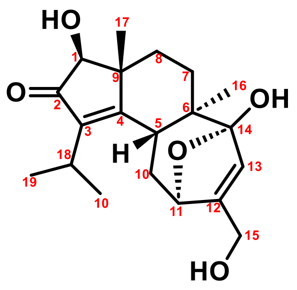

Neocyathin F (**3**)

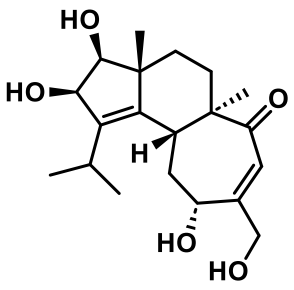

Neocyathin C (**4**)

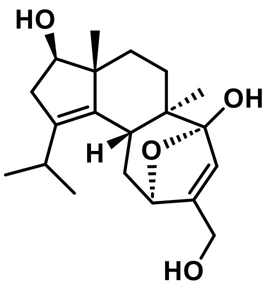

neocyathin E (**5**)

Table S14. Terpenoid biosynthesis related enzymes in *C. olla* SUT01 genome.

Type	Entry	UniportKB database-based annotations	E-value	Indentify	Species	Accession Number
STS	g12043.t1	Sesquiterpene synthase Agr1	$3e^{-49}$	33%	*Cyclocybe aegerita*	A0A5Q0QRJ3.1
	g12320.t1	Sesquiterpene synthase Agr8	$2e^{-61}$	42%	*Cyclocybe aegerita*	A0A5Q0QMX1.1
	g2256.t1	Sesquiterpene synthase Agr1	0.0	81%	*Cyclocybe aegerita*	A0A5Q0QRJ3.1
	g10143.t1	Germacrene-A synthase	0.0	80%	*Coprinopsis cinerea*	A8NE23.1
	g4632.t1	Sesquiterpene synthase COP6	$2e^{-14}$	38%	*Coprinopsis cinerea*	A8NCK5.1
	g13301.t1	Sesquiterpene synthase COP6	$2e^{-49}$	31%	*Coprinopsis cinerea*	A8NCK5.1
	g387.t1	Sesquiterpene synthase Agr1	$2e^{-30}$	26%	*Cyclocybe aegerita*	A0A5Q0QRJ3.1
	g6823.t1	Sesquiterpene synthase Agr8	$1e^{-130}$	54%	*Cyclocybe aegerita*	A0A5Q0QMX1.1
	g6831.t1	Sesquiterpene synthase Agr8	$3e^{-105}$	46%	*Cyclocybe aegerita*	A0A5Q0QMX1.1
	g6864.t1	Sesquiterpene synthase Agr1	$4e^{-27}$	27%	*Cyclocybe aegerita*	A0A5Q0QRJ3.1
SQS	g3759.t1	Squalene synthase	0	68%	*Ganoderma lucidum*	A0SJQ5.1
PSY	g1060.t1	NADH dehydrogenase (ubiquinone) complex I, assembly factor 6	$3e^{-53}$	34%	*Rattus norvegicus*	D3ZN43.1
Aromatic PT	g6976.t1	Tyrosine *O*-prenyltransferase tcpD	2e-34	29%	*Claviceps purpurea*	M1VV66.1
	g6980.t1	Tyrosine *O*-prenyltransferase sirD	4e-36	29%	*Plenodomus lingam*	Q6Q874.1
	g8775.t1	Tyrosine *O*-prenyltransferase sirD	6e-31	27%	*Plenodomus lingam*	Q6Q874.1

Table S15. Core genes with multi-domain in *C. olla* SUT01 genome.

Type	Entry	UniportKB database-based annotations	E-value	Identify	Species	Accession Number
NRPS-like-PKS	g10529.t1	PKS-NRPS hybrid synthetase hips	0.0	42%	*Neonothopanus nambi*	A0A3G9K3K9.1
	g391.t1	PKS-NRPS hybrid synthetase hips	0.0	44%	*Neonothopanus nambi*	A0A3G9K3K9.1
	g6859.t1	PKS-NRPS hybrid synthetase hips	0.0	44%	*Neonothopanus nambi*	A0A3G9K3K9.1
NRPS	g12999.t1	L-2-aminoadipate reductase	0.0	51%	*Schizosaccharomyces pombe*	P40976.3
	g12997.t1	L-2-aminoadipate reductase	0.0	51%	*Schizosaccharomyces pombe*	P40976.3
NRPS-like	g9064.t1	Nonribosomal peptide synthase-like enzyme 10	0.0	47%	*Heterobasidion annosum*	A0A1B1ZGB5.1
	g1352.t1	Nonribosomal peptide synthase-like protein cicB	$2e^{-62}$	32%	*Aspergillus nidulans*	A0A1U8QW91.1
	g1424.t1	Nonribosomal peptide synthase-like enzyme 10	0.0	34%	*Heterobasidion annosum*	A0A1B1ZGB5.1
	g6405.t1	Nonribosomal peptide synthase-like enzyme 10	0.0	36%	*Heterobasidion annosum*	A0A1B1ZGB5.1
	g3856.t1	Oxalate--CoA ligase	4e-40	29%	*Schizosaccharomyces pombe*	O74976.1
	g4533.t1	Nonribosomal peptide synthase-like enzyme 10	4e-118	34%	*Heterobasidion annosum*	A0A1B1ZGB5.1
	g4931.t1	Nonribosomal peptide synthase-like enzyme 11	0.0	43%	*Serpula lacrymans*	F8P9P5.2
	g4934.t1	Nonribosomal peptide synthase-like enzyme 11	0.0	43%	*Serpula lacrymans*	F8P9P5.2
	g11966.t1	Nonribosomal peptide synthase-like enzyme 10	7e-102	28%	*Heterobasidion annosum*	A0A1B1ZGB5.1
	g13235.t1	Nonribosomal peptide synthase-like enzyme 10	0.0	36%	*Heterobasidion annosum*	A0A1B1ZGB5.1
	g13408.t1	Nonribosomal peptide synthase-like enzyme 10	$4e^{-63}$	26%	*Heterobasidion annosum*	A0A1B1ZGB5.1
PKS	g9065.t1	Highly reducing polyketide synthase stpks1	0.0	50%	Strobilurus tenacellus	A0A384XH94.1
	g531.t1	Polyketide synthase 2	$7e^{-161}$	30%	Metarhizium majus	A0A0B4I1J8.1

Table S16. RIPP-like and NI-siderophore enzymes in *C. olla* SUT01 genome.

Type	Entry	UniportKB database-based annotations	E-value	Identify	Species	Accession Number
fungal-RiPP-like	g2528.t1	Phomopsin biosynthesis cluster protein Yc	$2e^{-18}$	27%	*Diaporthe leptostromiformis*	A0A8K1Y6E0.1
	g641.t1	Phomopsin biosynthesis cluster protein Yc	$2e^{-28}$	31%	*Diaporthe leptostromiformis*	A0A8K1Y6E0.1
	g2723.t1	Phomopsin biosynthesis cluster protein Yc	$3e^{-25}$	32%	*Diaporthe leptostromiformis*	A0A8K1Y6E0.1
	g3555.t1	Asperipin-2a biosynthesis cluster protein Y	$4e^{-18}$	33%	*Aspergillus flavus*	B8NCQ4.1
	g13242.t1	Phomopsin biosynthesis cluster protein Yc	$2e^{-20}$	35%	*Diaporthe leptostromiformis*	A0A8K1Y6E0
	g8684.t1	Phomopsin biosynthesis cluster protein Yc	$2e^{-22}$	31%	*Diaporthe leptostromiformis*	A0A8K1Y6E0
	g3557.t1	NA	NA	NA	NA	NA
NI-siderophore	g3057.t1	NRPS-independent siderophore synthetase rfs	$8e^{-128}$	37%	*Rhizopus delemar*	I1C129.1
	g3059.t1	NRPS-independent siderophore synthetase rfs	$2e^{-124}$	35%	*Rhizopus delemar*	I1C129.1

Descriptions | Graphic Summary | Alignments | Taxonomy

Sequences producing significant alignments Download Select columns Show 100

select all *100 sequences selected* GenBank Graphics Distance tree of results MSA Viewer

	Description	Scientific Name	Max Score	Total Score	Query Cover	E value	Per. Ident	Acc. Len	Accession
☑	Cyathus olla voucher 600 small subunit ribosomal RNA gene, partial sequence; internal transcribed spacer 1, 5.8...	Cyathus olla	436	436	100%	9e-118	100.00%	785	MH930129.1
☑	Cyathus olla strain Fe141F small subunit ribosomal RNA gene, partial sequence; internal transcribed spacer 1, 5....	Cyathus olla	436	436	100%	9e-118	100.00%	812	OP539090.1
☑	Cyathus hookeri voucher SWFC 20799 18S ribosomal RNA gene, partial sequence; internal transcribed spacer 1,...	Cyathus hookeri	436	436	100%	9e-118	100.00%	683	DQ463346.1
☑	Cyathus olla isolate iNaturalist # 153498241 small subunit ribosomal RNA gene, partial sequence; internal transcr...	Cyathus olla	431	431	100%	4e-116	99.58%	785	OR827172.1
☑	uncultured Agaricales genomic DNA sequence contains ITS1	uncultured Agari...	431	431	100%	4e-116	99.58%	384	OW844352.1
☑	Cyathus olla isolate 136 18S ribosomal RNA gene, partial sequence; internal transcribed spacer 1, 5.8S ribosoma...	Cyathus olla	431	431	100%	4e-116	99.58%	749	KC989088.1
☑	Cyathus olla voucher PDD-86833 small subunit ribosomal RNA gene, partial sequence; internal transcribed spac...	Cyathus olla	431	431	100%	4e-116	99.58%	761	MT444086.1
☑	Uncultured fungus isolate saf_F1244 5.8S ribosomal RNA gene, partial sequence; internal transcribed spacer 2, c...	uncultured fungus	431	431	100%	4e-116	99.58%	413	MW163873.1
☑	Cyathus olla voucher BPI 727227 18S ribosomal RNA gene, partial sequence; internal transcribed spacer 1, 5.8S...	Cyathus olla	431	431	100%	4e-116	99.58%	682	DQ463345.1
☑	Cyathus africanus voucher DAOM 200370 18S ribosomal RNA gene, partial sequence; internal transcribed space...	Cyathus africanus	425	425	100%	2e-114	99.15%	683	DQ463347.1
☑	Cyathus colensoi voucher DAOM 200423 18S ribosomal RNA gene, partial sequence; internal transcribed spacer...	Cyathus colensoi	422	422	100%	3e-113	98.74%	684	DQ463344.1
☑	Cyathus olla f. brodiensis voucher SWFC 21137 18S ribosomal RNA gene, partial sequence; internal transcribed ...	Cyathus olla f. br...	422	422	100%	3e-113	98.74%	684	DQ463343.1
☑	Cyathus hookeri voucher KA16-1024 internal transcribed spacer 1, partial sequence; 5.8S ribosomal RNA gene a...	Cyathus hookeri	420	420	100%	9e-113	98.73%	655	MK351663.1
☑	Cyathus jiayuguanensis voucher CLZhao 28026 internal transcribed spacer 1, partial sequence; 5.8S ribosomal ...	Cyathus jiayugua...	416	416	100%	1e-111	98.32%	774	OR554903.1
☑	Cyathus jiayuguanensis voucher CLZhao 28027 internal transcribed spacer 1, partial sequence; 5.8S ribosomal ...	Cyathus jiayugua...	416	416	100%	1e-111	98.32%	774	OR554902.1
☑	Cyathus africanus voucher CLZhao 27629 internal transcribed spacer 1, partial sequence; 5.8S ribosomal RNA g...	Cyathus africanus	416	416	100%	1e-111	98.32%	788	OR554897.1
☑	Cyathus africanus voucher CLZhao 27635 internal transcribed spacer 1, partial sequence; 5.8S ribosomal RNA g...	Cyathus africanus	416	416	100%	1e-111	98.32%	776	OR554896.1
☑	Cyathus africanus voucher CLZhao 27628 internal transcribed spacer 1, partial sequence; 5.8S ribosomal RNA g...	Cyathus africanus	416	416	100%	1e-111	98.32%	790	OR554895.1
☑	Cyathus hookeri strain L63 internal transcribed spacer 1, partial sequence; 5.8S ribosomal RNA gene and interna...	Cyathus hookeri	416	416	100%	1e-111	98.32%	763	KC005989.1
☑	Cyathus africanus strain L38 18S ribosomal RNA gene, partial sequence; internal transcribed spacer 1, 5.8S ribo...	Cyathus africanus	416	416	100%	1e-111	98.32%	763	JX103204.1

Figure S1. The ITS of *Cyathus olla* SUT01 was aligned to NCBI nr database.

RID: XBA7WBH701N.

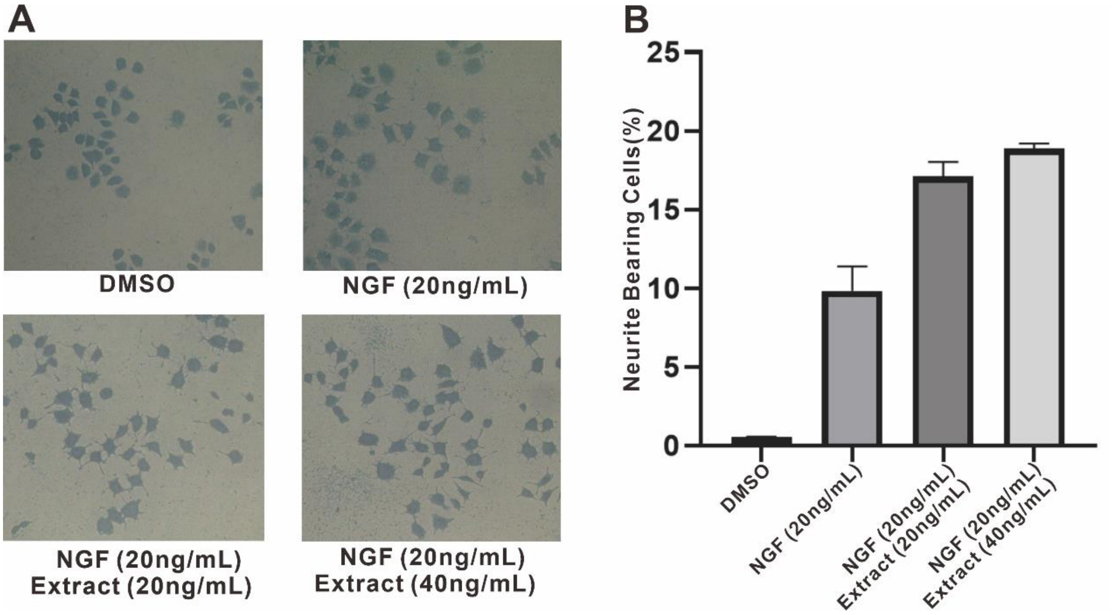

Figure S2. NGF-dependent promotional activity of crude extract of *C. olla* on rat pheochromocytoma PC12 cells.

Morphological characteristics (A) and promotion rate (B) of PC-12 cells treated with the crude extract of *C. olla* at different concentrations.

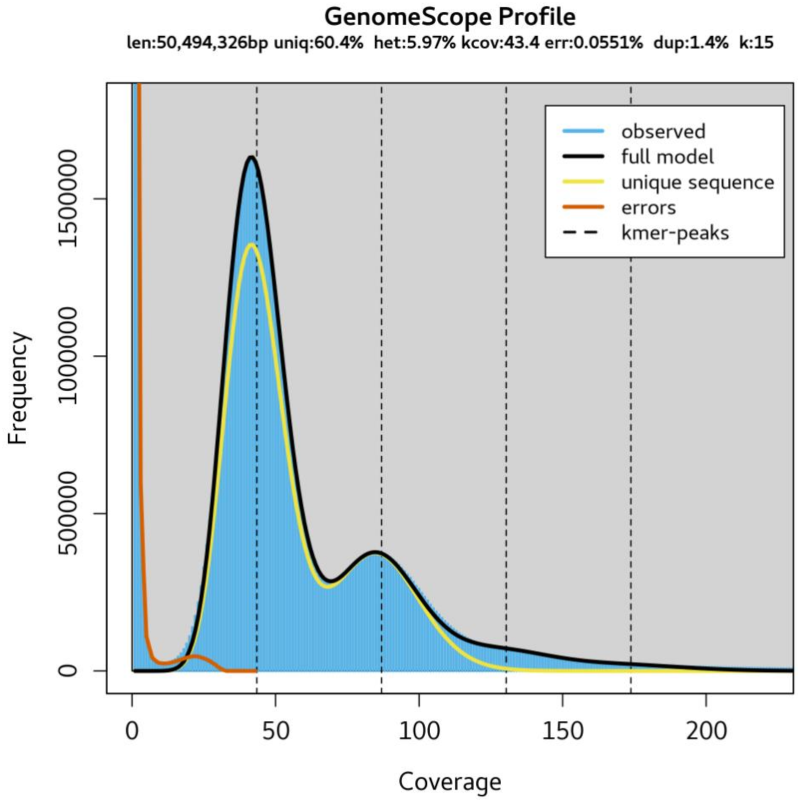

Figure S3. Kmer-Depth and Kmer Species-Frequency Distribution Plot.

The blue line represents the actual K-mer curve, the black line is the k-mer curve estimated by the model, the yellow line is the K-mer curve corresponding to the unique data, the red line represents the error curve due to sequencing errors, and the dashed line represents speculation K-mer peak.

Using the reads obtained by sequencing, K-mer-based analysis was used to estimate the genome size and heterozygosity. A K-mer refers to a sequence of K bp in length. Iteratively select a sequence of length K bases from a continuous sequence. If the length of the sequence is L and the length of the K-mer is K, then L-K+1 K-mers can be obtained. We take K-mers for the reads obtained by sequencing, and then count the frequency of each K-mer. According to the Lander_waterman algorithm, the genome size (G) satisfies the following formula:

$$C_{base} = C_{k-mer} \times \frac{L}{L-K+1}$$

$$G = \frac{n_{k-mer}}{C_{k-mer}} = \frac{n_{base}}{C_{base}}$$

C_{base} and $C_{k\text{-}mer}$ are the expected depth of coverage and K-mer, and nbase and nk-mer are the total number of bases and the total number of K-mers in the sequence. In the case of a certain amount of data, the depth frequency of K-mer is subject to Poisson distribution, so the peak of the K-mer depth frequency distribution is the corresponding depth, which is used as an estimate of the expected depth of K-mer.

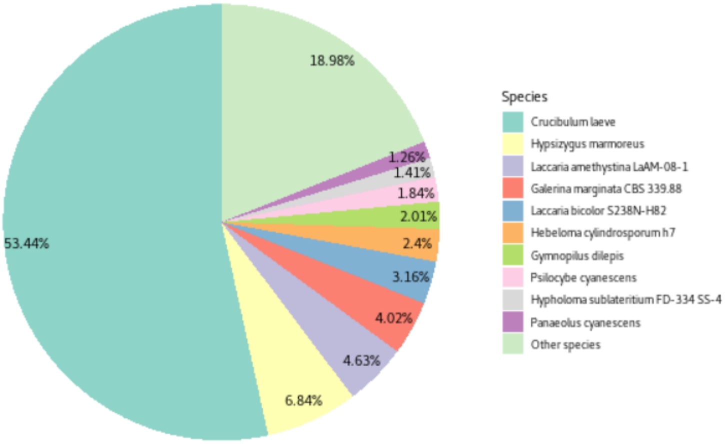

Figure S4. Species distribution map of Nr database alignment to sequences.

According to the results of the Nr library match, the top 10 species were counted and the rest were classified as other species, and the distribution of these species was mapped according to their proportion.

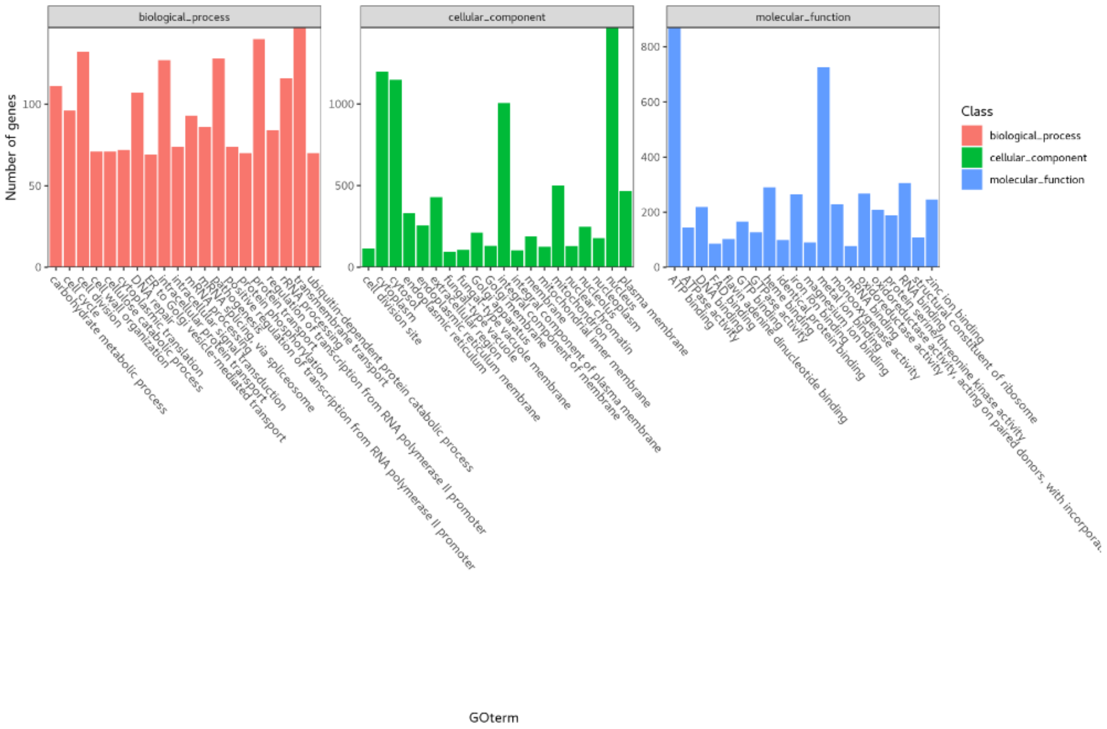

Figure S5. Statistical map of functional annotation classification based on GO database

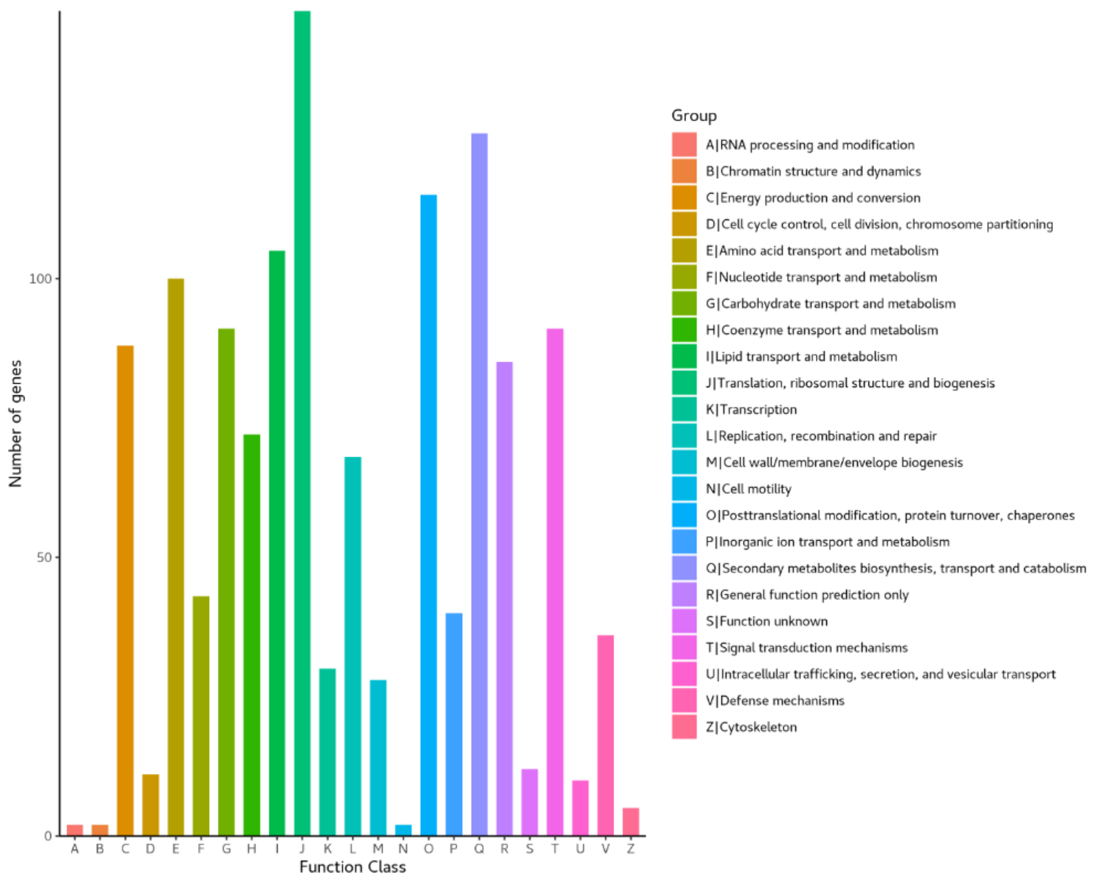

Figure S6. Statistical Chart of COG Functional Annotated Classification.

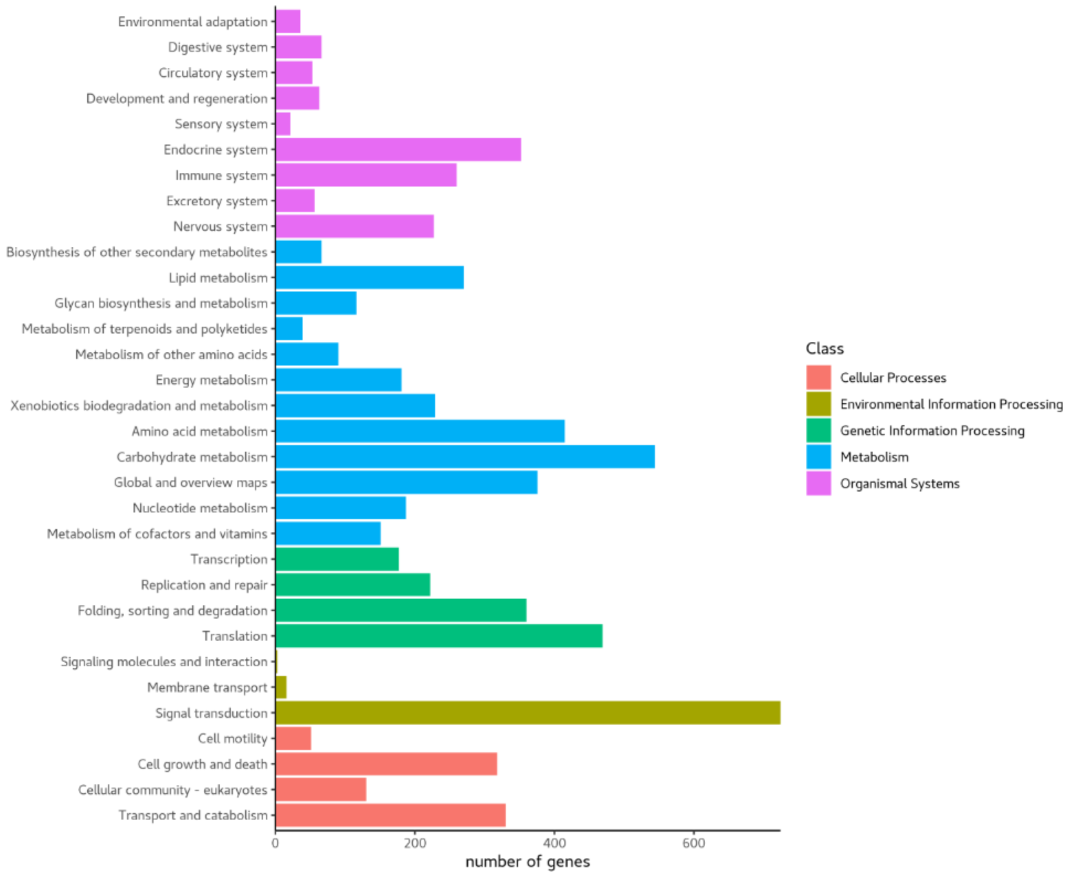

Figure S7. KEGG Pathway Functional Classification Chart.

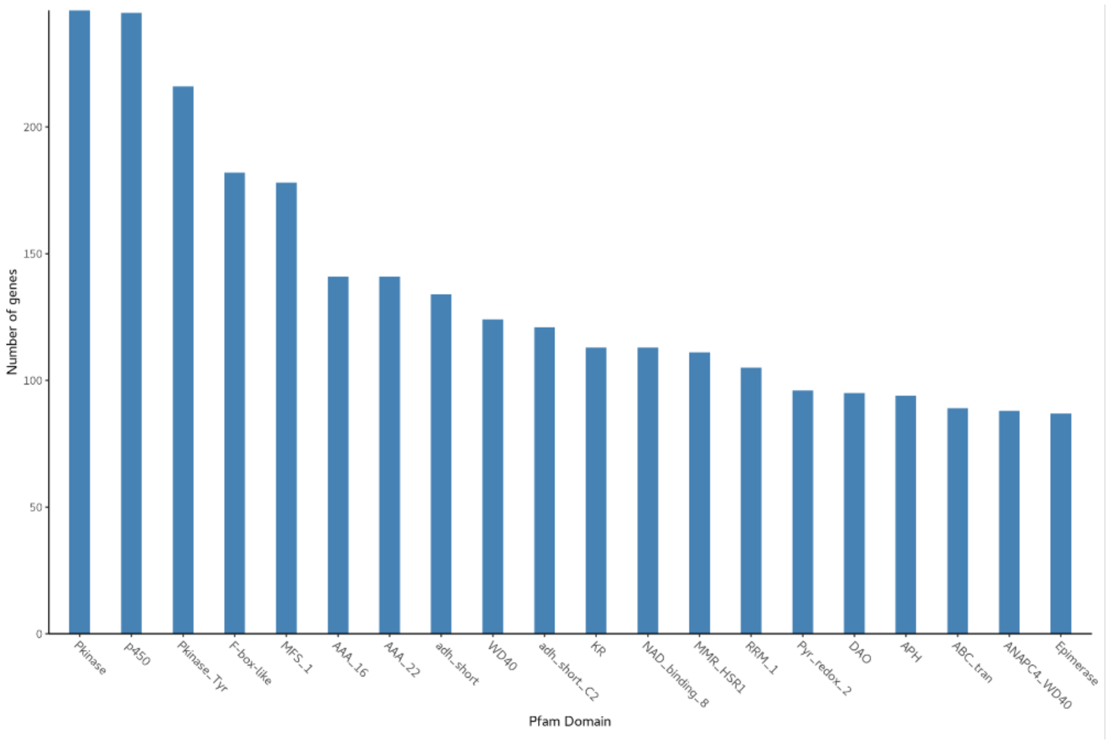

Figure S8. Domain annotation based on the Pfam database.

Statistical summaries of genes annotated for each domain are made and the top 20 most annotated domains are plotted.

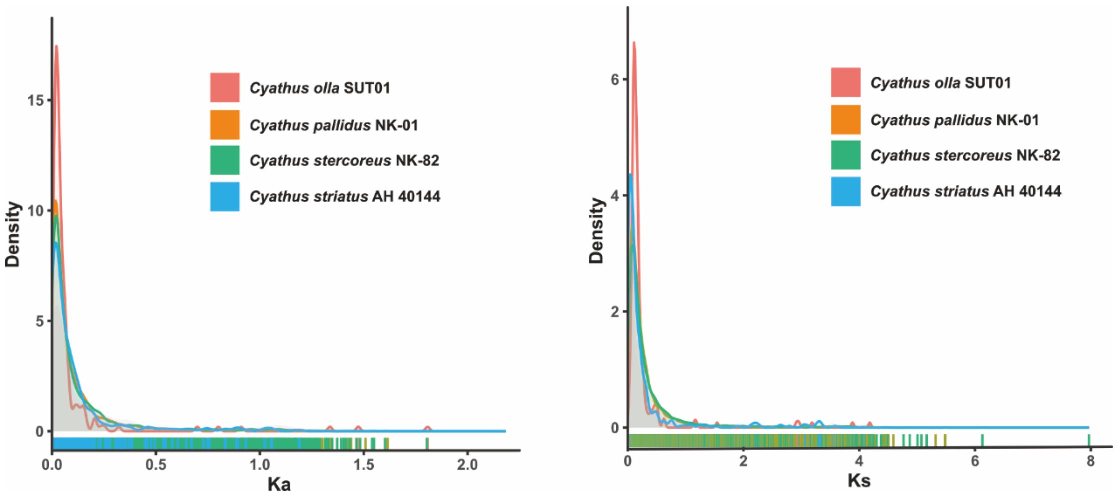

Figure S9. Ka and Ks comparisons of four *Cyathus* species.

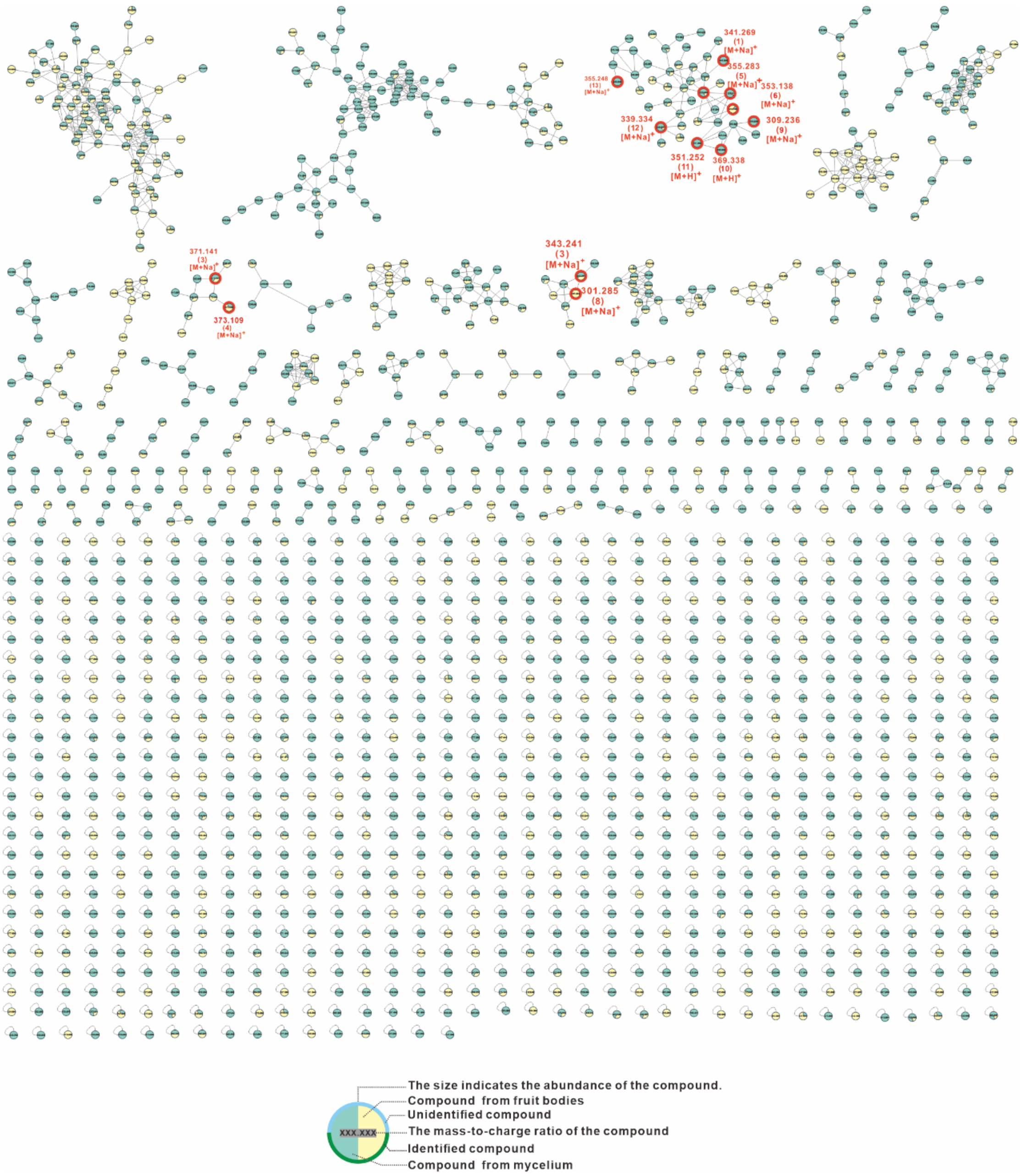

Figure S10. Molecular network analysis of metabolites from the mycelium and fruiting bodies of *C. olla* SUT01.

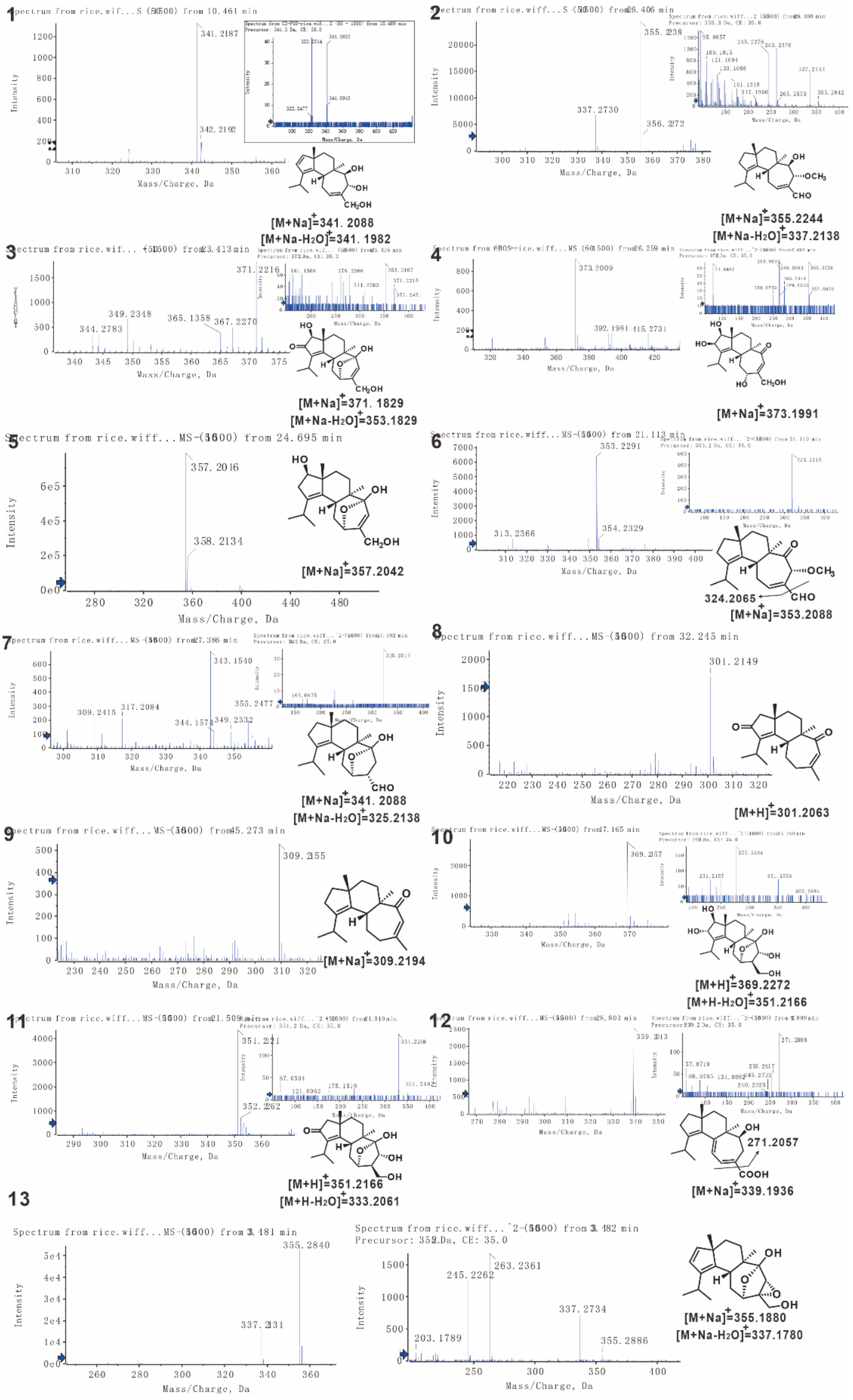

Figure S11. The LC-ESI-HRMS and LC-ESI-HRMS/MS spectrums of isolates from *C. olla* SUT01.

The mass spectrometry data were obtained in positive ion mode and numbers 1-13 correspond to compounds 1-12 one by one.

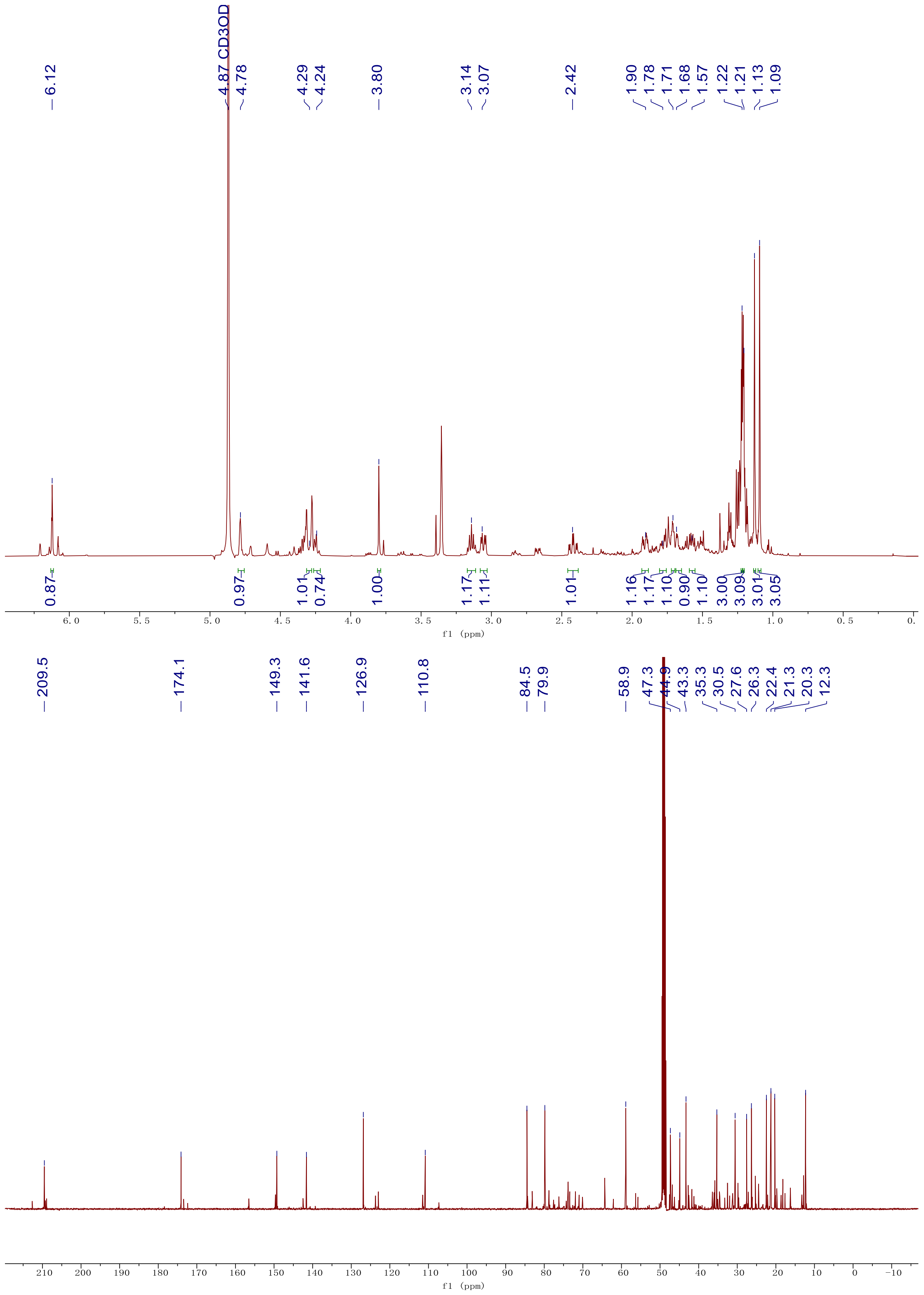

Figure S12. The ^{1}H and ^{13}C NMR spectra of Compound 3.

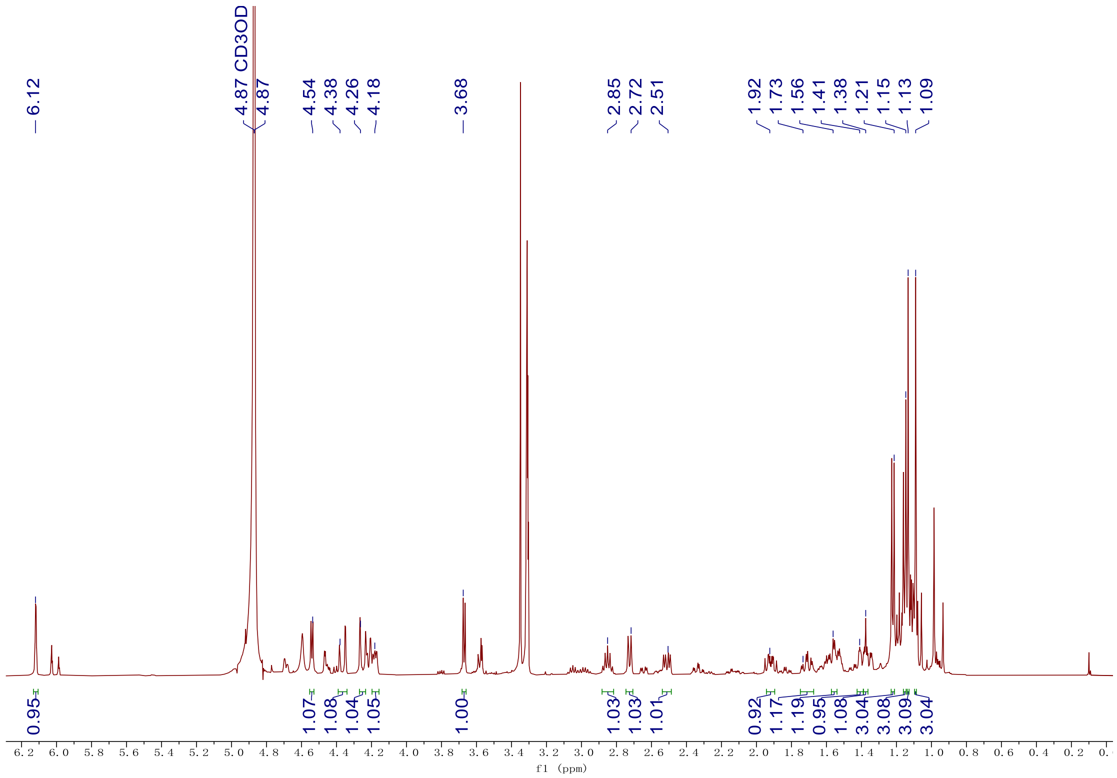

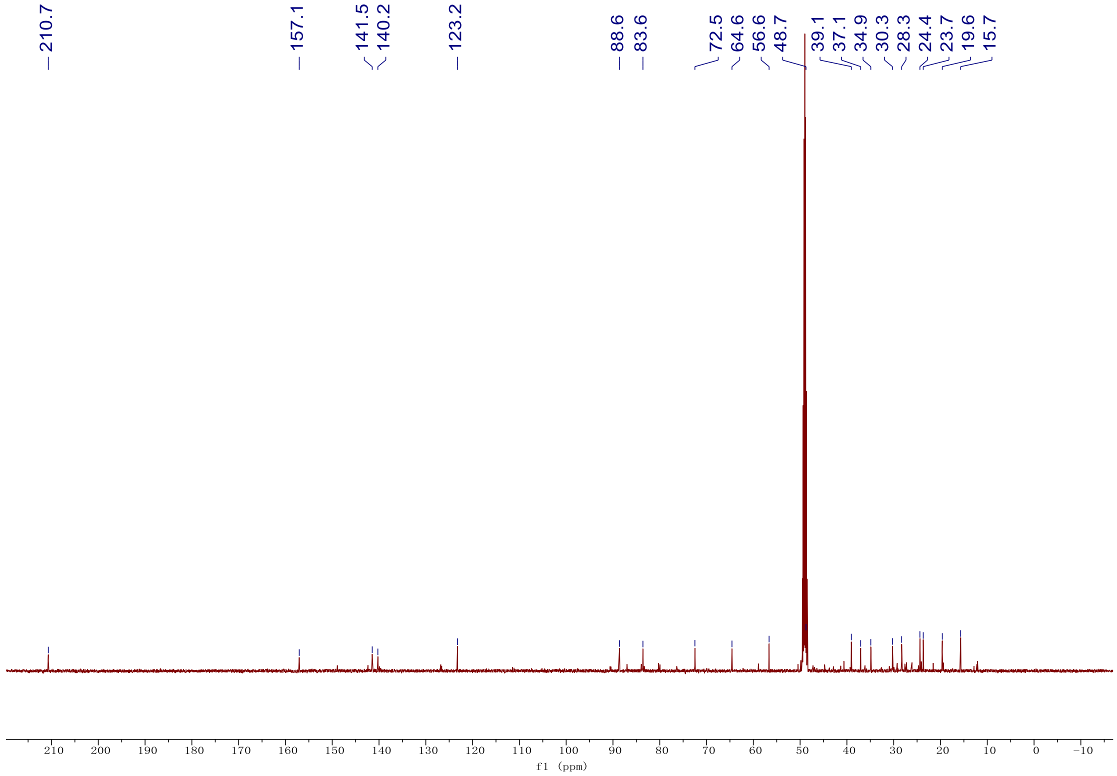

Figure S13. The ^{1}H and ^{13}C NMR spectra of Compound 4.

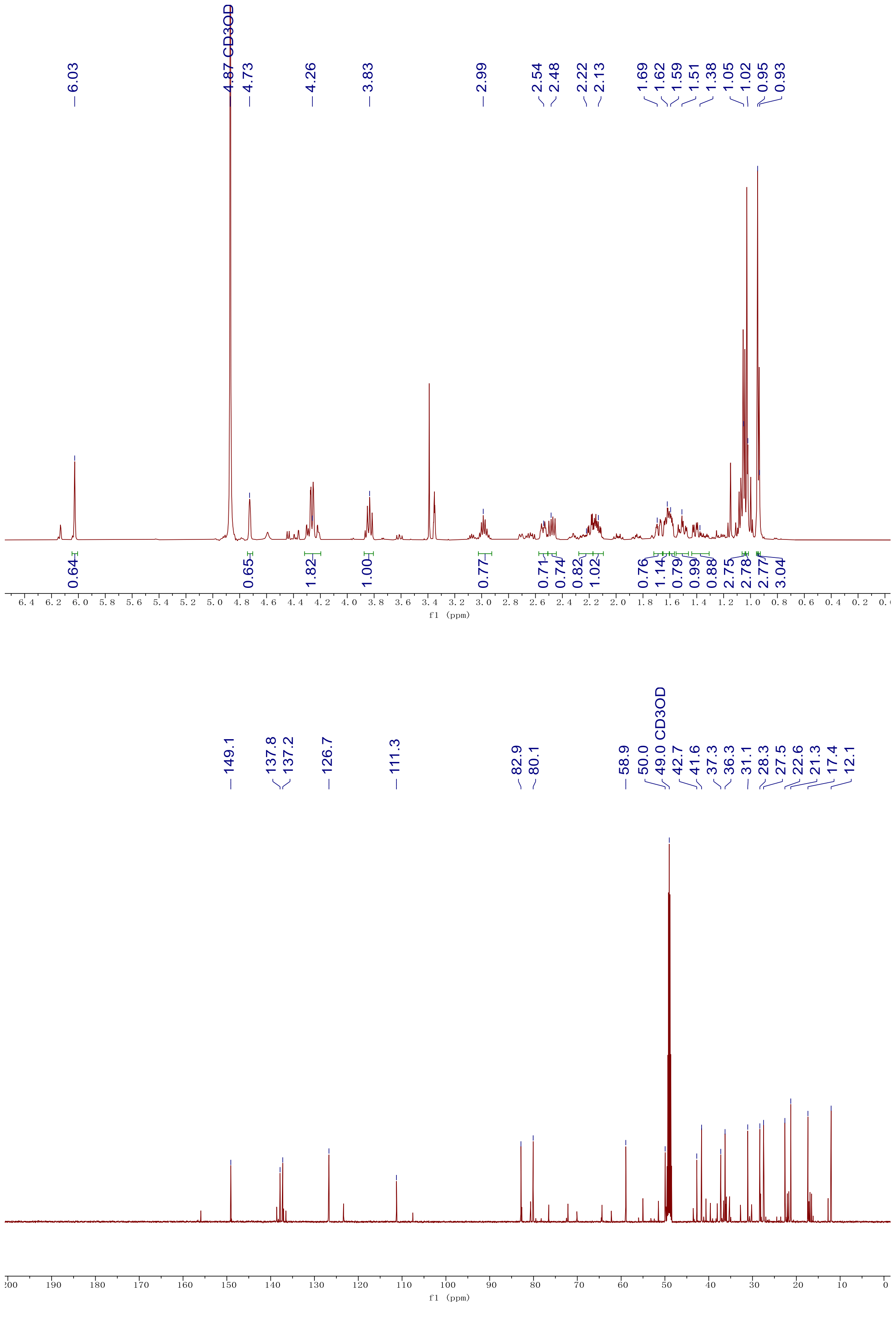

Figure S14. The ^{1}H and ^{13}C NMR spectra of Compound 5.

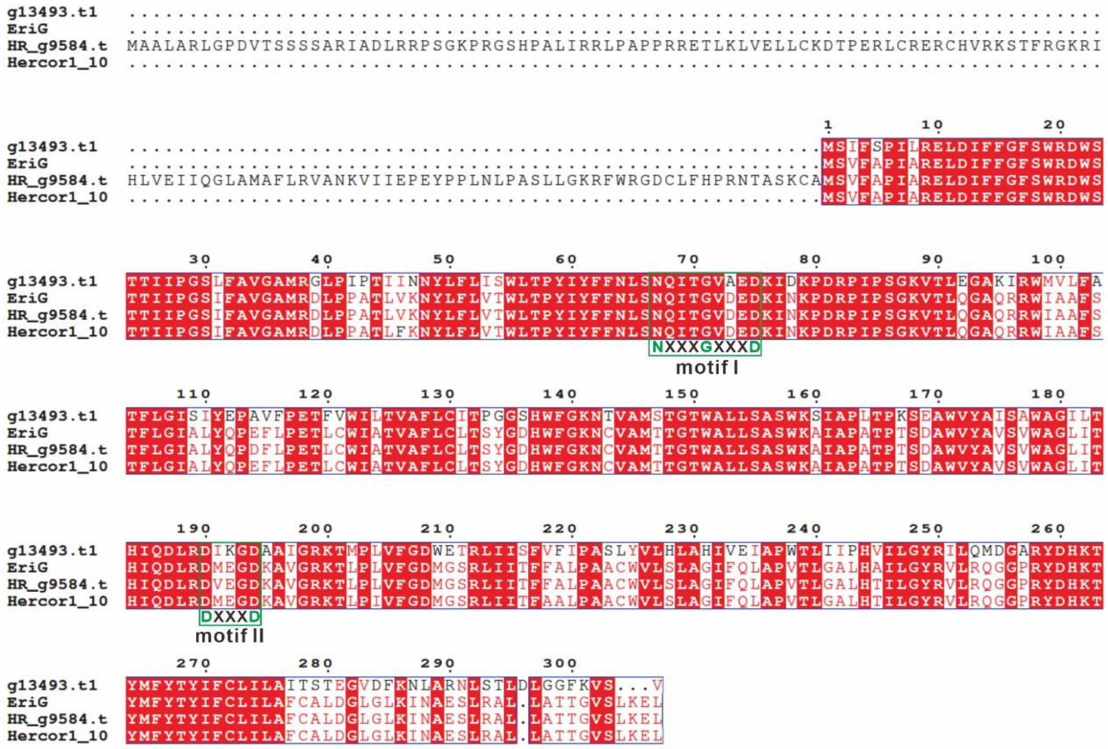

Figure S15. The two conserved motifs in proposed cyathane diterpene cyclase from *C. olla* SUT01.

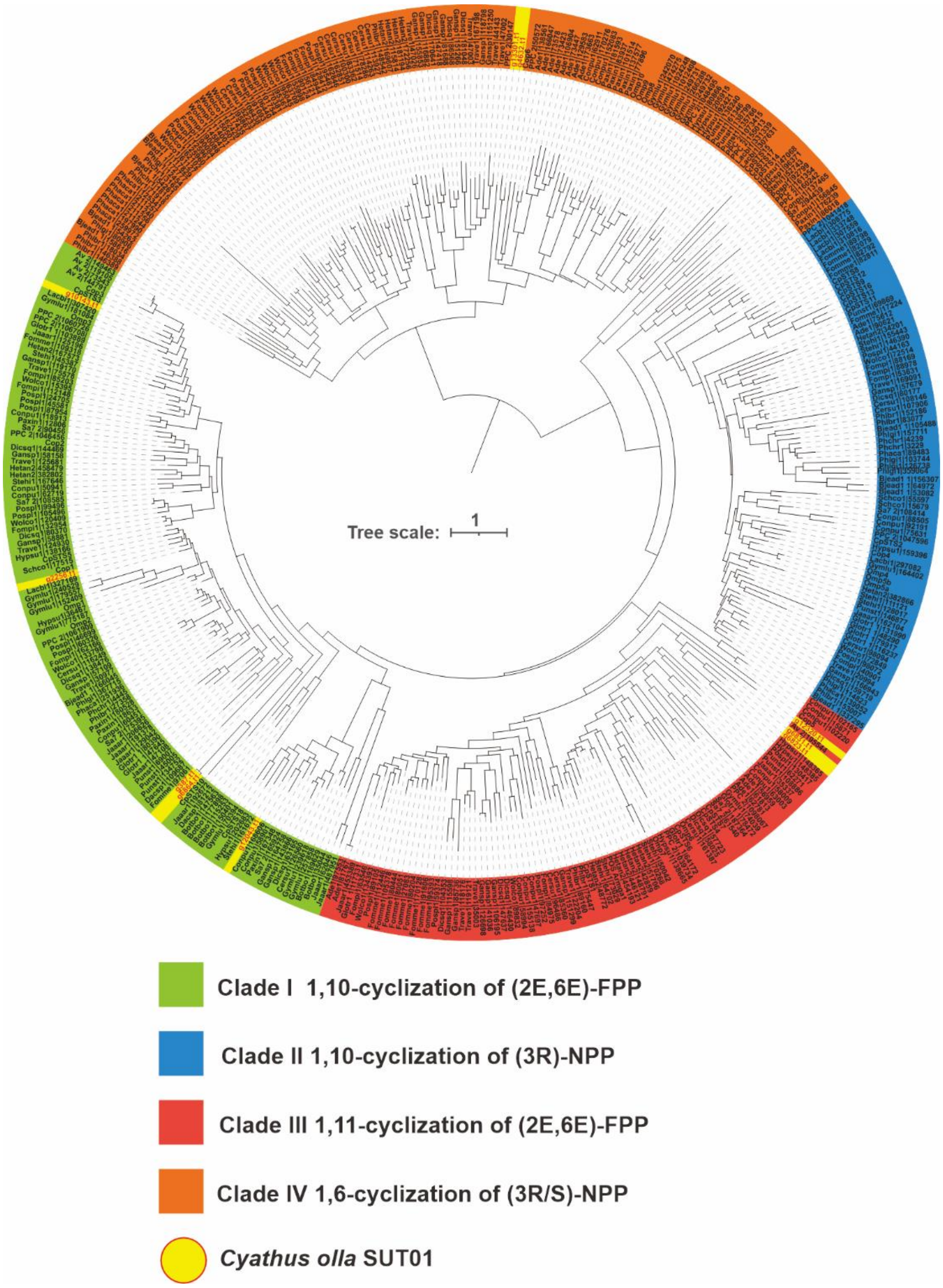

Figure S16. FPPs Cluster analysis of the strain *C. olla* SUT01 and other Basidiomycetes.

The identified FPP sequences used for clustering were obtained from a reported literature. Multiple sequence alignment and evolutionary tree construction were performed as described above. Multiple sequence pairs are implemented with mafft V7 .505[1] with parameters –maxiterate 1000 --localpair. The evolutionary tree was constructed by IQtreeV2.2.3[2] with the parameters -m MFP -bb 1000 -alrt 1000 -abayes -nt AUTO.

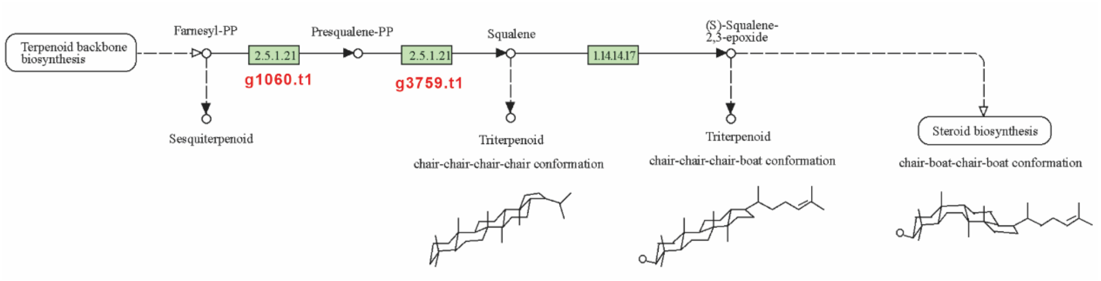

Figure S17. Annotation of the triterpenoid biosynthetic pathway of *C. olla* SUT01 using KAAS.

The genes with green background represent *C. olla* SUT01-derived genes annotated into the pathway, and proteins with red background represent specific *C. olla* SUT01-derived proteins involved in MVP. KAAS annotation is done by KEGG Automatic Annotation Server Ver. 2.1.

Reference

1. Katoh, K., Misawa, K., Kuma, K. i. and Miyata, T. 2002, MAFFT: a novel method for rapid multiple sequence alignment based on fast Fourier transform. *Nucleic acids research*, **30**, 3059-3066.
2. Minh, B. Q., Schmidt, H. A., Chernomor, O., et al. 2020, IQ-TREE 2: new models and efficient methods for phylogenetic inference in the genomic era. *Molecular biology and evolution*, **37**, 1530-1534.
